# Unresolved stalled ribosome complexes restrict cell-cycle progression after genotoxic stress

**DOI:** 10.1016/j.molcel.2022.01.019

**Published:** 2022-04-21

**Authors:** Mark Stoneley, Robert F. Harvey, Thomas E. Mulroney, Ryan Mordue, Rebekah Jukes-Jones, Kelvin Cain, Kathryn S. Lilley, Ritwick Sawarkar, Anne E. Willis

**Affiliations:** 1 MRC Toxicology Unit, University of Cambridge, Tennis Court Road, Cambridge CB2 1QR, UK; 2 Cambridge Centre for Proteomics, Cambridge Systems Biology Centre, Department of Biochemistry, University of Cambridge, Cambridge CB2 1QR, UK

**Keywords:** ribosome stalling, ribosome-associated quality control, RNA-binding protein, ASC-1 complex, RNA damage, ultraviolet light, cell-cycle arrest

## Abstract

During the translation surveillance mechanism known as ribosome-associated quality control, the ASC-1 complex (ASCC) disassembles ribosomes stalled on the mRNA. Here, we show that there are two distinct classes of stalled ribosome. Ribosomes stalled by translation elongation inhibitors or methylated mRNA are short lived in human cells because they are split by the ASCC. In contrast, although ultraviolet light and 4-nitroquinoline 1-oxide induce ribosome stalling by damaging mRNA, and the ASCC is recruited to these stalled ribosomes, we found that they are refractory to the ASCC. Consequently, unresolved UV- and 4NQO-stalled ribosomes persist in human cells. We show that ribosome stalling activates cell-cycle arrest, partly through ZAK-p38^MAPK^ signaling, and that this cell-cycle delay is prolonged when the ASCC cannot resolve stalled ribosomes. Thus, we propose that the sensitivity of stalled ribosomes to the ASCC influences the kinetics of stall resolution, which in turn controls the adaptive stress response.

## Highlights

• The ASCC splits many types of stalled ribosome, but some are refractory to the ASCC
• ASCC-resistant stalled ribosomes are caused by bulky adduct damage within the mRNA
• Failure of ASCC to resolve stalled ribosomes promotes a prolonged cell-cycle arrest
• Unresolved stalled ribosomes activate ZAK/p38^MAPK^ signaling leading to G2 arrest

## Graphical abstract

**Figure:**
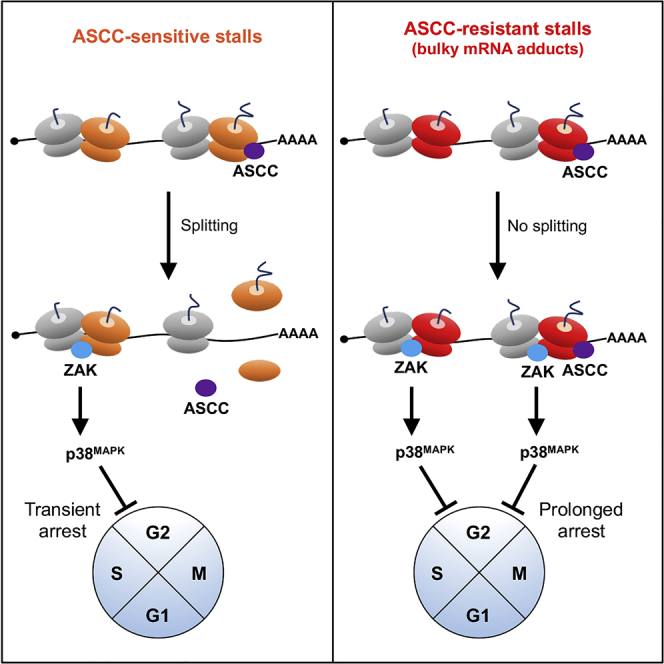

## Teaser

Stoneley et al. reveal that although the ribosome splitting activity of the ASCC resolves many ribosomes stalled during translation, ribosomes stalled by bulky nucleobase mRNA modifications are refractory to the ASCC. ASCC-resistant stalled ribosomes are long lived in the cell and stimulate prolonged cell-cycle arrest through the ZAK/p38^MAPK^ signaling axis.

## Introduction

When a ribosome stalls at a problematic region of the mRNA during translation, a potentially toxic truncated polypeptide is generated. However, the surveillance mechanism known as ribosome-associated quality control (RQC) eliminates the polypeptide within the stalled ribosome (Joazeiro, 2019; Sitron and Brandman, 2020). Failure of RQC results in proteotoxicity and neurodegeneration, highlighting the consequences of unresolved stalled ribosomes (Choe et al., 2016; Chu et al., 2009).

RQC occurs in three sequential phases. First, a collision between a stalled ribosome and the next upstream ribosome creates a collided disome that is recognized and ubiquitylated by ZNF598 (Garzia et al., 2017; Ikeuchi et al., 2019; Juszkiewicz et al., 2018; Juszkiewicz and Hegde, 2017; Matsuo et al., 2017; Sundaramoorthy et al., 2017). Subsequently, the ASC-1 complex (ASCC) splits the stalled 80S ribosome into ribosomal subunits (Hashimoto et al., 2020; Juszkiewicz et al., 2020; Matsuo et al., 2020). Finally, the nascent peptidyl-tRNA within the 60S ribosome is ubiquitylated by the RQC complex, leading to polypeptide degradation by the proteasome (Bengtson and Joazeiro, 2010; Brandman et al., 2012; Lyumkis et al., 2014; Shao et al., 2013, 2015).

Our understanding of RQC comes from studying the translation of repetitive sequences, such as poly(A), that are problematic for the ribosome. These sequences adopt a conformation in the A-site of the ribosome that is incompatible with efficient decoding (Chandrasekaran et al., 2019; Tesina et al., 2020), which results in ribosome stalling, ribosome collisions, and clearance of the nascent polypeptide by RQC (D'Orazio et al., 2019; Sitron et al., 2017). Although repetitive tracts have been crucial in unraveling the mechanism of RQC, other features within an mRNA also impede the translating ribosome (Arpat et al., 2020; Han et al., 2020; Meydan and Guydosh, 2020; Simms et al., 2014; Yan et al., 2019). Environmental stress, including ultraviolet light (UV), alkylative and oxidative agents, causes chemical damage to RNA that can hinder the translating ribosome (Hudson and Zaher, 2015; Simms et al., 2014; Wu et al., 2020; Yan et al., 2019). Ribosome stalling has been observed after UV irradiation or alkylation damage (Wu et al., 2020; Yan and Zaher, 2021), and ZNF598-dependent ubiquitylation of ribosomal proteins eS10 and uS10 occurs in response to UV, methylation, or oxidation stress (Garshott et al., 2020; Yan et al., 2019; Yan and Zaher, 2021). Nevertheless, our knowledge of the surveillance mechanisms and physiological outcomes induced by RNA damage is currently incomplete.

The RNA-helicase ASCC3 splits ribosomes stalled within the coding region of an mRNA (Juszkiewicz et al., 2020; Matsuo et al., 2020). We found that ultraviolet light-B (UVB) or 4-nitroquinoline 1-oxide (4NQO) stress induced ribosome stalling, which was associated with the accumulation of the ASCC on the stalled ribosomes and increased ASCC3 RNA binding. However, although the ASCC recognizes UVB- and 4NQO-stalled ribosomes, it cannot resolve them, resulting in long-lived unresolved stalled ribosomes. Failure of the ASCC to resolve stalled ribosomes causes prolonged cell-cycle arrest through the ZAK-p38^MAPK^ signaling axis, revealing a link between the longevity of stalled ribosomes and the adaptive stress response.

## Results

### Bulky nucleic acid damage induces ASCC3 RNA binding

Cytoplasmic gene expression is controlled through networks of RNA-protein interactions (Tenenbaum et al., 2011). To understand the role of these networks in the cellular UV stress response, we analyzed the changes in cytoplasmic RNA-protein interactions that occur after UVB irradiation.

RNA-interactome capture (RIC) identifies high-confidence RNA-protein interactions *in vivo* (Castello et al., 2013). Proteins are crosslinked to their RNA ligands *in vivo*, isolated through their attached RNA, and the RNA-binding proteins (RBPs) are analyzed by mass spectrometry (Figure S1A). We used fractionation coupled with RIC (Piñeiro et al., 2018) to catalog the cytoplasmic RBPs of MCF10A cells by comparing the proteins in RIC eluates from untreated or crosslinked cells (Figure S1B). Proteins enriched in the crosslinked sample were classified as RBPs, leading to the identification of 270 putative cytoplasmic RBPs (Table S1). RIC was then performed on control and UVB-treated cells (Figure S1C), which identified 6 proteins with potentially increased association with RNA after UVB irradiation (Table S2). We focused on ASCC3, which had the largest increase in RNA interaction after UVB stress and was identified as a cytoplasmic RBP.

**Figure 1:**
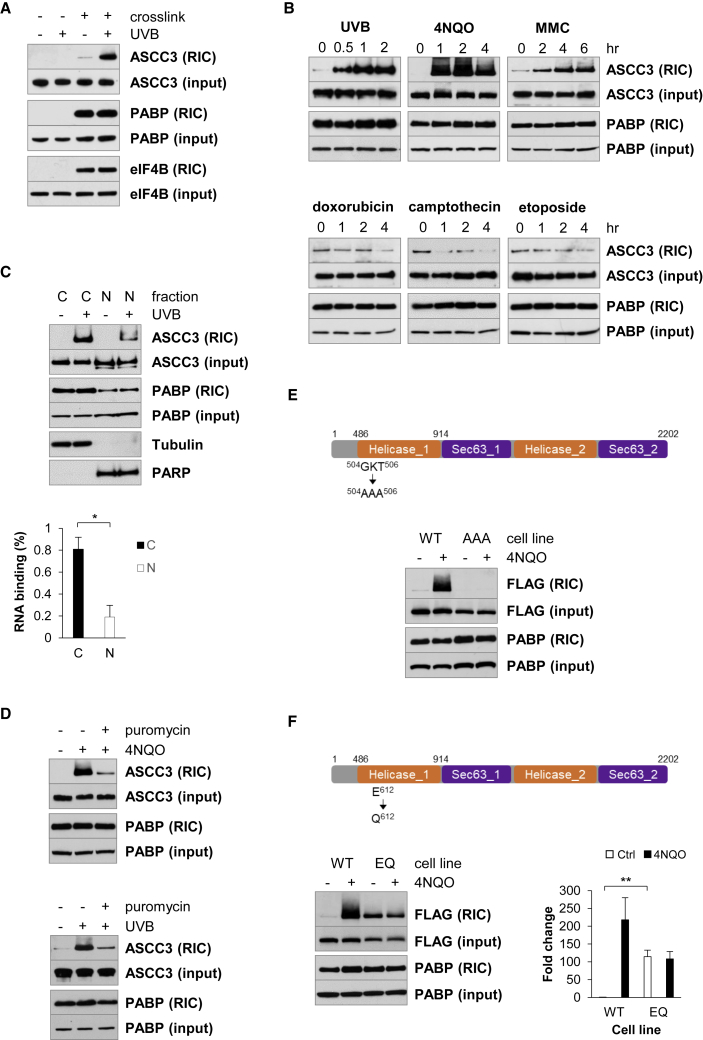
Bulky nucleic acid damage induces ASCC3 RNA binding (A) ASCC3 RNA binding using RIC in mock and UVB-irradiated MCF10A cells. (B) ASCC3 RNA binding in MCF10As exposed to nucleic acid damaging agents. (C) ASCC3 RNA binding in cytoplasmic (C) and nuclear (N) fractions after UVB treatment. Error bars represent the SD. ∗p < 0.05. (D) The effect of puromycin pre-treatment on ASCC3 RNA binding after UVB or 4NQO exposure. (E and F) *In vivo* RNA binding of ASCC3-F (WT) and ASCC3-F^AAA^ (AAA) in (E) or ASCC3-F (WT) and ASCC3-F^E612Q^ (EQ) in (F) in response to 4NQO. Error bars represent the SD. ∗∗p < 0.01. See also Figure S1.

To confirm that UVB stress increases the interaction of ASCC3 with RNA, RIC was performed on mock and UVB-treated cells followed by western analysis. ASCC3 was only recovered in the crosslinked RIC samples, and there was a 46-fold (p = 0.02) increase in ASCC3 isolated by RIC after UVB (Figure 1A). Neither ASCC3 in the input samples nor the capture of control RBPs (PABP and eIF4B) in the RIC assay was affected by UVB treatment. Taken together, these data confirm that the interaction between ASCC3 and RNA increases markedly after UVB stress in MCF10A cells. Furthermore, UVB stimulated ASCC3 RNA binding to a similar extent in transformed cells (Figure S1D).

ASCC3 RNA binding increased in response to treatments that cause bulky adduct damage to nucleic acid, UVB, ultraviolet light-C (UVC), 4NQO, and mitomycin C (MMC) (Figures 1B and S1E). However, reagents that create DNA strand breaks, doxorubicin, camptothecin, and etoposide, did not increase ASCC3 RNA binding (Figure 1B), suggesting that bulky nucleobase damage may be the stimulus for increased ASCC3 RNA binding.

Although ASCC3 plays a role in the nuclear processes of transcription and DNA repair (Boeing et al., 2016; Dango et al., 2011; Jung et al., 2002), RIC analysis combined with subcellular fractionation showed that 80% of the ASCC3 RNA binding occurred in the cytoplasm (Figure 1C). Moreover, treating cells with puromycin prior to UVB or 4NQO prevented the increase in ASCC3 RNA binding (Figure 1D). Therefore, ASCC3 RNA interaction is associated with a cytoplasmic function that requires active mRNA translation.

ASCC3 contains two Ski2-like helicase domains, but only the N-terminal domain is active (Jia et al., 2020). Since RNA helicases are ATP-gated RBPs, we investigated the link between ASCC3 RNA binding and helicase activity.

The GKT motif in the N-terminal helicase domain of ASCC3 is essential for ATP binding and RNA-helicase activity (Jia et al., 2020). Therefore, we mutated the N-terminal GKT motif and determined the effect on ASCC3 RNA binding *in vivo* (Figure 1E). 4NQO stress produced a large increase in wild-type (WT) ASCC3 RNA binding (ASCC3-F), whereas the GKT mutant protein (ASCC3-F^AAA^) failed to bind RNA under these conditions. Thus, ASCC3 RNA binding is entirely dependent on ASCC3 ATPase activity.

ASCC3 also contains a DExD/H motif in its active helicase domain (Henn et al., 2012). The glutamic acid residue of the DExD/H motif is involved in ejecting phosphate after ATP hydrolysis, which promotes the release of the RNA ligand. Mutation of this residue to a glutamine slows the rate of phosphate release and can convert a helicase into an ATP-gated RNA clamp (Xiol et al., 2014). We tested the *in vivo* RNA binding of ASCC3 with a glutamine at position 612 (ASCC3-F^E612Q^) (Figure 1F). Compared with the WT protein, the interaction of ASCC3-F^E612Q^ with RNA increased by 114-fold in the untreated cells (Figure 1F, lanes 1 and 3). Thus, the E612Q mutation slows the release of RNA from the helicase, resulting in accumulation of ASCC3-F^E612Q^ on its RNA ligand.

Taken together, these data indicate that ASCC3 RNA binding is intrinsically linked to RNA-helicase activity.

### The ASCC accumulates on UV- and 4NQO-stalled ribosomes

ASCC3 has been reported to dissociate ribosomes during the translation-dependent process of RQC (Juszkiewicz et al., 2020; Matsuo et al., 2017, 2020; Sitron et al., 2017). Furthermore, genotoxic stress can cause ribosome stalling during mRNA translation (Wu et al., 2020; Yan et al., 2019; Yan and Zaher, 2021). Because ASCC3 RNA binding is dependent on mRNA translation, we reasoned that ASCC3 RNA binding may be due to UV- and 4NQO-induced ribosome stalling.

**Figure 2:**
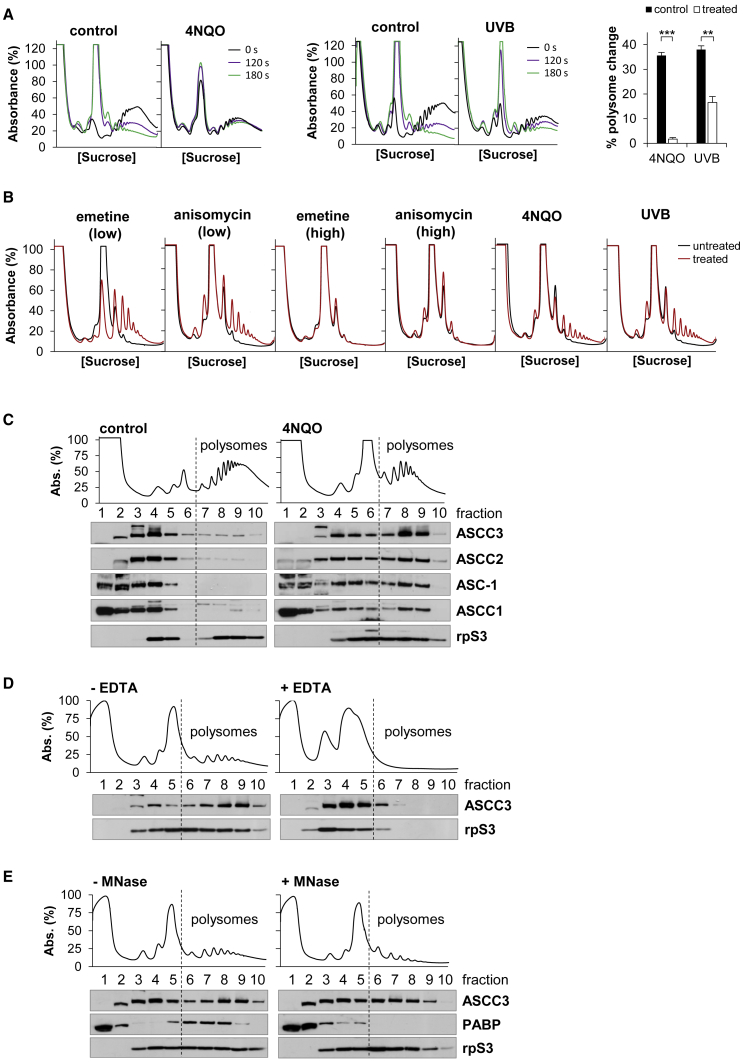
The ASCC accumulates on UVB- and 4NQO-stalled ribosomes (A) Harringtonine run-off assays to measure the rate of polysome loss in HeLa cells treated with 4NQO or UVB. Error bars represent the SD. ∗∗p < 0.01, ∗∗∗p < 0.001. (B) Micrococcal nuclease assay to measure ribosome collisions in HeLa cells treated with 2-μM emetine (low), 0.2-μM anisomycin (low), 300-μM emetine (high), 75-μM anisomycin (high), 4NQO, or UVB for 15 min. (C) Distribution of the ASCC proteins after SDGC of lysates prepared from control or 4NQO-treated cells. (D and E) ASCC3 distribution after SDGC of lysates prepared from cells exposed to 4NQO. Lysates were treated with EDTA (+EDTA) in (D) or MNase (+MNase) in (E). See also Figure S2.

Ribosomes stalled by UV or 4NQO would reduce the elongation rate of ribosomes upstream of the stall site. The ribosome transit rate of an mRNA population can be measured using a harringtonine run-off assay (Ingolia et al., 2011). Treatment of HeLa cells with harringtonine resulted in a time-dependent reduction of polysomes and an increase in 80S ribosomes (Figure 2A). However, when the cells were exposed to 4NQO or UVB, polysome loss induced by harringtonine was decreased by 23.7 and 2.3-fold, respectively (Figure 2A). Therefore, ribosome run-off is slower after UVB or 4NQO treatment of cells.

Reduced ribosome run-off after 4NQO and UVB could be due to either the presence of stalled ribosomes on the mRNA or to a decrease in the global rate of ribosome elongation. Stalled ribosomes result in ribosome collisions directly upstream of the stall site (Ikeuchi et al., 2019; Juszkiewicz et al., 2018; Simms et al., 2017). We used an assay that detects ribosome collisions *in vivo* to determine if UVB or 4NQO cause ribosome stalling (Juszkiewicz et al., 2018). Micrococcal nuclease (MNase) digests the mRNA of polysomes between the ribosomes, thereby releasing 80S ribosomes. However, when ribosome collisions occur, the inter-ribosomal mRNA of the collided ribosomes is protected from MNase digestion (Ikeuchi et al., 2019; Juszkiewicz et al., 2018), and MNase-resistant polysomes (MNase-RPs) are released (Figure S2A).

Polysomes from untreated cells were converted to 80S ribosomes with some residual disomes and trisomes after digestion with MNase. Furthermore, an 8-fold increase in the MNase concentration did not collapse these remaining disomes and trisomes (Figure S2B), suggesting that there is a basal level of ribosome collisions in the cell (Arpat et al., 2020; Han et al., 2020; Meydan and Guydosh, 2020). Emetine and anisomycin, two translation elongation inhibitors (TEIs) known to cause ribosome stalling (Juszkiewicz et al., 2018), were tested to confirm that increased ribosome collisions can be detected in this assay. After cells were exposed to a sub-saturating concentration of the TEIs, there was a substantial increase in the number of MNase-RPs, indicating that low TEI concentrations stalled ribosomes and caused collisions (Figure 2B). In contrast, when all ribosomes were stalled using high concentrations of the TEIs, ribosome collisions did not increase above the level in untreated cells (Figure 2B). Thus, the MNase assay identifies ribosome stalling through ribosome collisions. Treatment of cells with either 4NQO or UVB resulted in a sizable increase in MNase-RPs, confirming that both stresses stimulate ribosome stalling (Figure 2B).

To further investigate the link between ASCC3 and RQC after genotoxic stress, the interaction between ASCC3 and the translating ribosomes was analyzed using sucrose density gradient centrifugation (SDGC). The majority of ASCC3 did not associate with polysomes in control cells, whereas upon 4NQO treatment, there was a 6.4-fold (p < 0.001) increase of ASCC3 on the polysomes (Figure 2C). ASCC3 forms a complex with ASCC2, ASCC1, and ASC-1 (Hashimoto et al., 2020; Juszkiewicz et al., 2020), which also accumulated on the polysomes after 4NQO exposure (Figure 2C). UVB irradiation also caused a comparable shift of the ASCC on to polysomes (Figure S2C). ASCC3 relocated with the ribosomes to the sub-polysomal region of the gradient when polysomes extracted from 4NQO-treated cells were dissociated with EDTA (Figure 2D) or when translation was inhibited with puromycin prior to 4NQO treatment (Figure S2D), confirming that the ASCC associates with polyribosomes.

Formaldehyde crosslinking was used to establish that the ASCC associates with polysomes *in vivo*. Thus, when cells were not crosslinked, the harsher lysis conditions in these experiments disrupted the interaction between the ASCC and polysomes (Figure S2E). However, *in vivo* formaldehyde crosslinking of the cells prior to lysis revealed the accumulation of ASCC on the polysomes upon 4NQO treatment (Figure S2E). Therefore, 4NQO stress results in accumulation of the ASCC on polysomes *in vivo*.

To assess whether ASCC3 associates with stalled ribosomes, extracts prepared from 4NQO treated cells were digested with MNase, which yields polysomes composed of stalled and collided ribosomes. ASCC3 remained on the MNase-RPs after nuclease digestion (Figure 2E). In contrast, PABP relocated from the polysomes to the mRNP region of the gradient, confirming that the mRNA was completely digested (Figure 2E).

Taken together, these data indicate that UVB and 4NQO cause ribosome stalling, which in turn recruits the ASCC to stalled ribosomes and stimulates ASCC3 RNA binding.

### UVB- and 4NQO-stalled ribosomes are long lived in the cell

**Figure 3:**
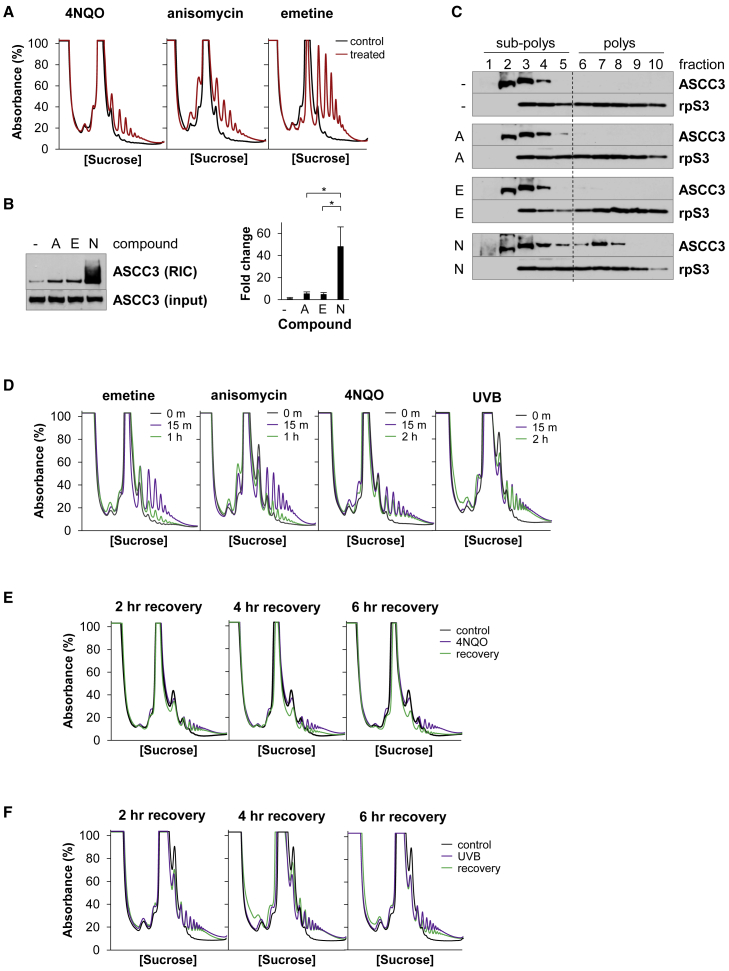
UVB- and 4NQO-stalled ribosomes persist in the cell (A) Ribosome collisions in HeLa cells treated with 4NQO, 0.6-μM anisomycin, or 0.6-μM emetine for 1 h. (B) ASCC3 RNA binding in HeLa cells treated with anisomycin (A), emetine (E), or 4NQO (N) as above. Error bars represent the SD. ∗p < 0.05. (C) ASCC3 distribution after SDGC of lysates prepared from cells treated as in (A). (D) Kinetic analysis of collided ribosomes in cells treated with 0.2-μM emetine or anisomycin, 4NQO, or UVB. (E and F) Collided ribosomes in HeLa cells recovering from exposure to 4NQO in (E) or UVB in (F).

We investigated whether TEIs, which also stimulate ribosome stalling, have similar effects on the ASCC as UVB or 4NQO. The concentration of 4NQO, emetine, and anisomycin was optimized to produce approximately equivalent ribosome stalling 1 h after exposure (Figure 3A). RIC analysis revealed that anisomycin and emetine treatment stimulated ASCC3 RNA binding, but far less effectively than 4NQO (Figure 3B). Furthermore, neither TEI caused ASCC3 to accumulate on polysomes (Figure 3C). These observations suggest that UVB- and 4NQO-stalled ribosomes differ from those induced by TEIs.

The previous observations could be explained if UVB- or 4NQO-stalled ribosomes are more stable in cells than TEI-stalled ribosomes. Hence, we compared the rate at which the different stalled ribosomes are resolved *in vivo*. Substantial quantities of MNase-RPs were detected in cells 15 min after TEI exposure and these collided ribosomes were greatly reduced after 1 h, suggesting that TEI-stalled ribosomes were disassembled within this time (Figure 3D). MNase-RPs also increased markedly after cells were exposed to UVB or 4NQO, but even 2 h after UVB or 4NQO stress, there was little change in the number of collided ribosomes (Figure 3D). A more extensive kinetic analysis showed that collided ribosomes were reduced after the cells had recovered for 4 or 6 h after 4NQO or UVB, respectively (Figures 3E and 3F). Thus, 4NQO- and UVB-stalled ribosomes are eventually removed from the cell, but they are more long lived compared with those induced by TEIs.

### The ASCC cannot resolve UVB- or 4NQO-stalled ribosomes

**Figure 4:**
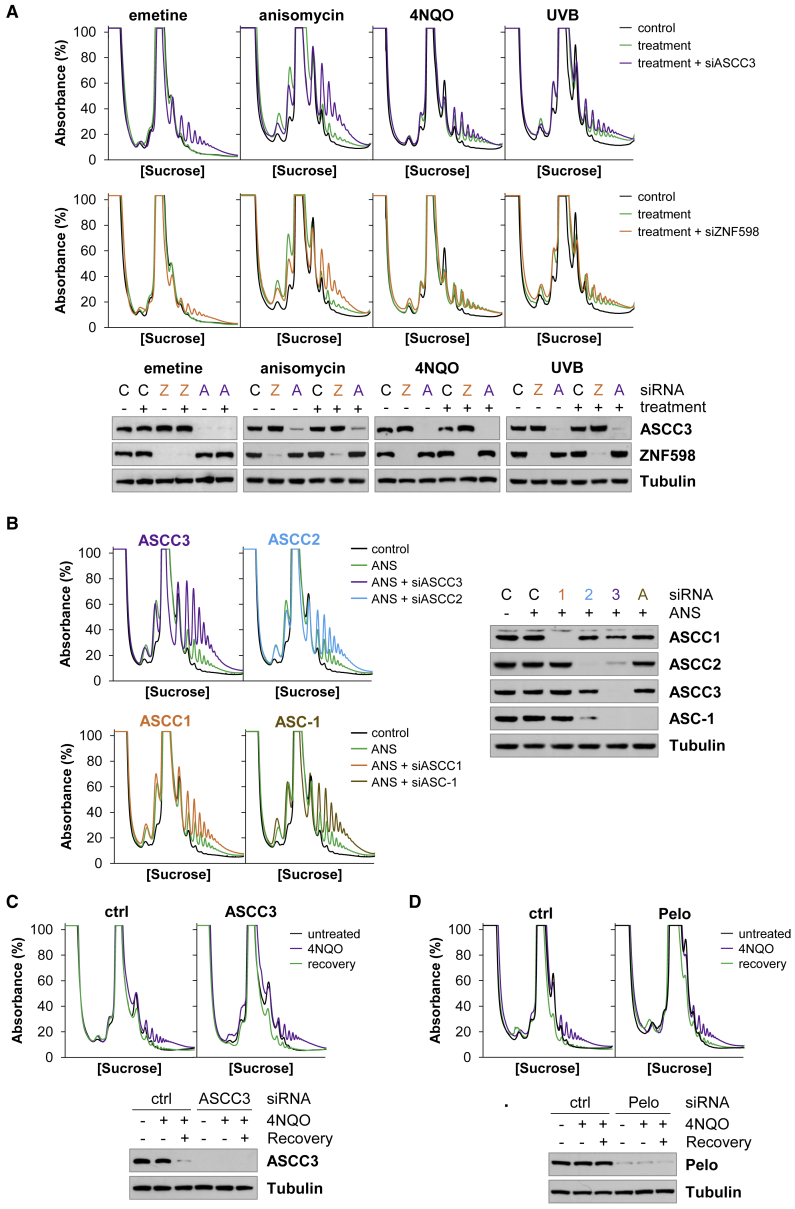
UV- and 4NQO-stalled ribosomes are resistant to the ASCC (A) The effect of ASCC3 or ZNF598 depletion on the loss of collided ribosomes 1 h after treatment with emetine, anisomycin, 4NQO, or UVB. ASCC3 (A) and ZNF598 (Z) were efficiently depleted compared with the control (C). (B) The effect of ASCC3, ASCC2, ASCC1, or ASC-1 depletion on the loss of collided ribosomes 1 h after treatment with anisomycin. ASCC1 (1), ASCC2 (2), ASCC3 (3), or ASC-1 (A) were efficiently depleted compared with the control (C). (C and D) ASCC3 depletion in (C) or Pelo depletion in (D) has no effect on the loss of 4NQO-stalled ribosomes. See also Figure S3.

The rapid loss of TEI-induced ribosome collisions (Figure 3D) could be due to the resolution of stalled ribosomes by RQC. To test this hypothesis, we analyzed the effect of the RQC factors, ASCC3 and ZNF598, on TEI-induced ribosome collisions. Depletion of ASCC3 or ZNF598 led to a considerable increase in the number of collided ribosomes 1 h after cells were exposed to the TEIs (Figure 4A). Collided ribosomes did not accumulate in untreated cells depleted of ASCC3 or ZNF598, confirming that the additional MNase-RPs are due to the combined effect of depletion and drug exposure (Figure S3A). Therefore, the resolution of ribosomes stalled by the TEIs requires ASCC3 and ZNF598.

Since the entire ASCC is recruited to stalled ribosomes (Figure 2), we assessed the impact of each of the ASCC proteins on the resolution of anisomycin-stalled ribosomes. Compared with control cells, there was an increase in MNase-RPs in the ASCC3, ASCC2, ASCC1, and ASC-1 depleted cells, suggesting that each member of the ASCC affects the resolution of anisomycin-stalled ribosomes (Figure 4B). However, the magnitude of this effect differs between the complex members. In contrast, depletion of the ribosome splitting factor Pelo did not prevent the loss of collided ribosomes after anisomycin treatment (Figure S3B). Thus, in agreement with a recent study, we find that the ASCC and not Pelo splits internally stalled ribosomes (Juszkiewicz et al., 2020).

Depletion of ASCC3 or ZNF598 had little effect on ribosome collisions 1 h after treatment of cells with 4NQO or UVB, confirming that 4NQO- and UVB-stalled ribosomes are resistant to the ASCC and RQC at this time (Figure 4A). 4NQO-stalled ribosomes are completely removed from the cell after a 6-h recovery (Figure 3E), but neither ASCC3 nor Pelo depletion prevented this loss of 4NQO-stalled ribosomes (Figures 4C and 4D). Therefore, ribosomes stalled by 4NQO or UVB are resistant to the ASCC and are eventually dealt with by an ASCC and Pelo-independent mechanism.

### ASCC-resistant stalled ribosomes are caused by mRNA damage

Covalent bonds can form between RNA and protein or RNA and RNA in response to UV irradiation (Wurtmann and Wolin, 2009). In addition, 4NQO can crosslink nucleic acid to protein by producing reactive oxygen species (ROS) (Groehler et al., 2018). Therefore, the inability of the ASCC to resolve UVB- or 4NQO-stalled ribosomes could be due to covalent linkage of the ribosome and the mRNA, or of the ribosomal subunits.

**Figure 5:**
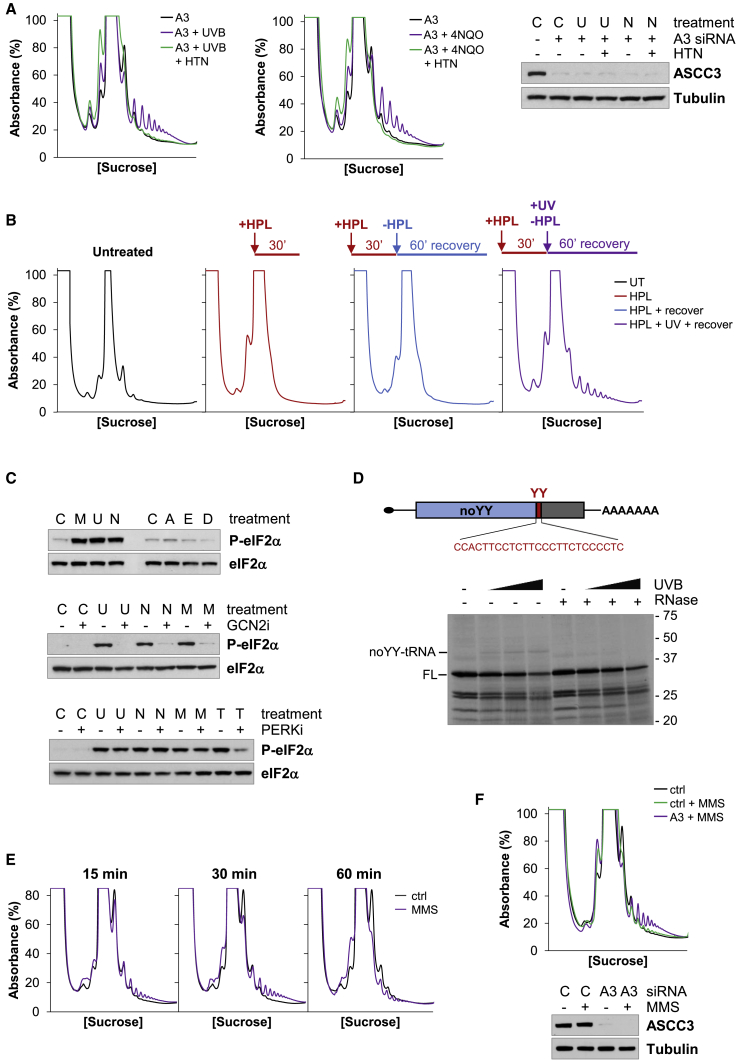
UVB and 4NQO induce ribosome stalling by damaging mRNA (A) Harringtonine run-off of UVB (U)- or 4NQO (N)-stalled ribosomes in ASCC3 (A3)-depleted cells. (B) UVB irradiation of HeLa cells, in which translation is blocked with hippuristanol (HPL), results in ribosome stalling when mRNA translation resumes. (C) The effect of MMS (M), UVB (U), 4NQO (N), anisomycin (A), emetine (E), or didemnin B (D) on eIF2α phosphorylation (upper). GCN2i prevents eIF2α phosphorylation in response to UVB, 4NQO, or MMS (middle). PERKi prevents eIF2α phosphorylation in response to thapsigargin (T), but not UVB, 4NQO, or MMS (lower). (D) Schematic of the UVPD reporter mRNA (noYY sequence, blue; pyrimidine tract, red; remaining coding sequence, gray). *In vitro* translation of untreated and UVB-damaged UVPD reporter mRNA. The noYY peptidyl-tRNA (noYY-tRNA) and full-length protein (FL) are indicated. (E) Ribosome collisions after MMS treatment. (F) Depletion of ASCC3 (A3) prevents the resolution of MMS-stalled ribosomes. See also Figure S4.

To determine whether ASCC-refractory-stalled ribosomes are caused by ribosome-mRNA crosslinking, we inhibited translation with harringtonine in cells treated with UVB or 4NQO and measured the run-off of collided ribosomes. We observed a time-dependent loss of collided ribosomes in UVB and 4NQO-treated cells after harringtonine addition (Figure S4A). Ribosome collisions in UVB and 4NQO-treated cells returned to the basal level after 40 min of ribosome run-off in both ASCC3 replete and depleted cells (Figures 5A and S4A). Moreover, the loss of collided ribosomes occurs before 4NQO- or UVB-stalled ribosomes are resolved in the cell (Figures 3E and 3F). Thus, UVB- and 4NQO-stalled ribosomes are not covalently linked to the mRNA.

We reasoned that if UVB-stalled ribosomes are due to crosslinking of the translating ribosomal subunits, stalls will not form if the cells are UV irradiated when there are no translating ribosomes and then mRNA translation is allowed to resume. However, if UVB-stalled ribosomes are due to mRNA or aminoacyl-tRNA (aa-tRNA) damage, ribosomes should stall on the mRNA when they recommence translation. Hippuristanol (HPL), a reversible eIF4A inhibitor, blocked translation initiation in HeLa cells and mRNA translation partially recovered after the cells were removed from HPL (Figure S4B). UVB irradiation of cells immediately before releasing them from HPL caused collided ribosomes to accumulate when mRNA translation resumed (Figure 5B). In contrast, there was no increase in ribosome collisions in cells released from HPL without UVB stress (Figure 5B). UVB-stalled ribosomes that form after cells are released from a translation block persist for at least 4 h in the cell and are ASCC-resistant (Figures S4C and S4D). Therefore UVB-induced ASCC-resistant stalls are caused by damage to mRNA or aa-tRNA.

Methyl methanesulfonate (MMS) methylates mRNA causing ribosome stalling that activates the integrated stress response. Methylated nucleobases in the mRNA impede tRNA selection, stalling the ribosome with an empty A-site, which stimulates eIF2α phosphorylation by the GCN2 kinase (Yan and Zaher, 2021). We found that similar to MMS, both UVB and 4NQO stimulated eIF2α phosphorylation, but anisomycin, emetine, and didemnin B, which stall ribosomes with an occupied A-site, did not have this effect (Figure 5C). Furthermore, inhibition of GCN2 prevented eIF2α phosphorylation after UVB, 4NQO, and MMS, whereas PERK inhibition did not have this effect (Figure 5C). GCN2-dependent phosphorylation of eIF2α after UVB or 4NQO supports the hypothesis that these stresses damage aa-tRNA or mRNA, which hinders tRNA recognition.

Ribosome stalled by damaged aa-tRNA with impaired codon recognition would be similar to ribosomes stalled by limiting aa-tRNA levels. Therefore, we explored whether the ASCC can resolve ribosomes stalled by aa-tRNA deficiency. Depletion of an amino acid reduces the cellular level of cognate aa-tRNAs, causing ribosome pausing at the corresponding codon. Ribosome pausing triggers a GCN2-mediated feedback mechanism that inhibits mRNA translation initiation and prevents ribosome collisions (Darnell et al., 2018; Wu et al., 2020). Thus, we saw an increase in ribosome collisions in cells deprived of arginine and lysine when GCN2 was inhibited, indicating that ribosomes are stalled under these conditions (Figure S4E). Ribosome collisions induced by aa-tRNA deficiency increased after ASCC3 depletion (Figure S4F). Therefore, ASCC-resistant ribosome stalling cannot be due to aa-tRNA damage that hinders codon recognition.

Damaged aa-tRNA could also prevent the accommodation of the aa-tRNA into the A-site. Didemnin B is an inhibitor of eEF1A that locks the aa-tRNA-eEF1A complex in the A-site with the aa-tRNA in a pre-accomodated state (Shao et al., 2016). Depletion of ASCC3 increased ribosome collisions in didemnin-B-treated cells, indicating that the ASCC can resolve ribosomes stalled with the aa-tRNA in a pre-accommodated state in the ribosome (Figure S4G). Finally, although it is possible that damaged aa-tRNA could interfere with the peptidyl transferase reaction, this would not lead to ASCC-resistant stalls because ribosomes stalled by anisomycin, which blocks the peptidyl transferase reaction, can be resolved by the ASCC (Figure 4). Taken together, these data suggest that damaged aa-tRNA is unlikely to generate ASCC-resistant stalls. Therefore, we propose that damaged mRNA is most likely responsible for ASCC-resistant ribosome stalling.

UV is known to introduce photolesions within an mRNA, particularly at pyrimidines (Wurtmann and Wolin, 2009). To determine if UV lesions in the mRNA can cause ribosome stalling, we programmed a translating extract with UVB-irradiated *in-vitro*-transcribed mRNA. Damaging the mRNA with UVB decreased the synthesis of the full-length protein (FL) and resulted in the production of several truncated polypeptides, consistent with ribosome stalling on UVB-damaged mRNA (Figure S4H). We suspected that UV-induced pyrimidine dimers (UVPDs) would be particularly problematic for the translating ribosome. Hence, we designed a UVPD reporter mRNA with a region after the initiation codon with no adjacent pyrimidines (noYY), followed by a short pyrimidine tract (YY) (Figure 5D). UVPDs will form in the YY region of the reporter after UVB irradiation and translation of the damaged reporter will produce noYY peptidyl-tRNAs if UVPDs cause ribosome stalling. *In vitro* translation of UVB-damaged UVPD reporter mRNA produced a radiolabeled product of approximately 42 kDa that was not present in the extracts programmed with undamaged mRNA (Figure 5D, noYY-tRNA). As the predicted mass of the noYY polypeptide is 24 kDa, the migration of this product is consistent with noYY peptidyl-tRNA. Furthermore, the product cannot be detected when the extracts are digested with RNase, confirming that it is noYY peptidyl-tRNA (Figure 5D). Therefore, UVPDs within an mRNA induce ribosome stalling.

We then asked whether any damage to the mRNA’s nucleobases would prevent ASCC-dependent ribosome splitting by analyzing the resolution of ribosomes stalled at sites of methylation damage in the mRNA (Yan and Zaher, 2021). MMS caused a transient accumulation of collided ribosomes in HeLa cells and the loss of these collided ribosomes 1 h after treatment was attenuated in ASCC3 depleted cells (Figures 5E and 5F). Thus, mRNA damage by MMS produces stalled ribosomes that are susceptible to the ASCC. Hence, it seems likely that specific base modifications introduced into the mRNA by UV or 4NQO, such as pyrimidine dimers, prevent the resolution of stalled ribosomes by the ASCC.

### Unresolved stalled ribosomes cause cell-cycle arrest

**Figure 6:**
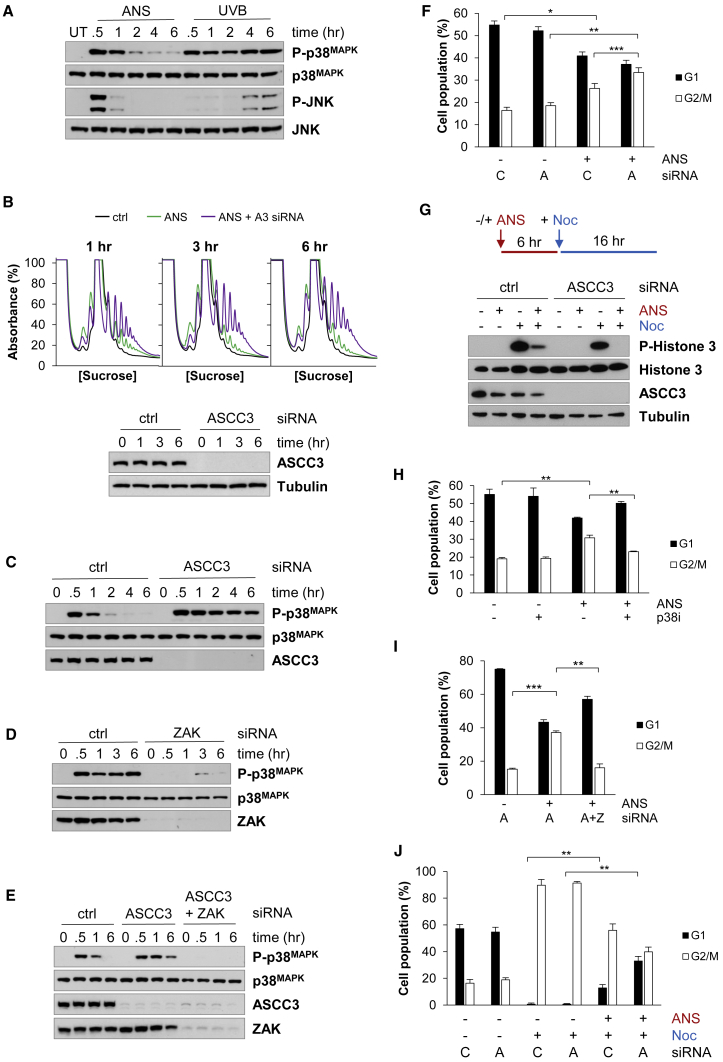
Unresolved stalled ribosomes cause cell-cycle arrest (A) Phosphorylation of p38^MAPK^ and JNK after treatment with anisomycin (ANS) or UVB. (B) Collided ribosomes at 1, 3, and 6 h after treatment with anisomycin in control or ASCC3-depleted cells. (C) ASCC3 depletion causes prolonged p38^MAPK^ phosphorylation in response to anisomycin. (D and E) p38^MAPK^ phosphorylation after UVB treatment in (D) or after anisomycin treatment of ASCC3-depleted cells in (E) depends on ZAK. (F) Cell-cycle distribution in control (C) and ASCC3-depleted cells (A) after treatment with anisomycin. Error bars represent the SD. ∗p < 0.05, ∗∗p < 0.01, ∗∗∗p < 0.001. (G) The effect of anisomycin treatment in control and ASCC3-depleted cells on the G2 to M-phase transition measured by trapping the cells in mitosis with nocodazole (Noc). (H and I) The accumulation of ASCC3-depleted cells in G2 after anisomycin treatment is prevented by pre-exposure to a p38^MAPK^ inhibitor (p38i) in (H) or depletion of ZAK in (I). ∗∗p < 0.01, ∗∗∗p < 0.001. (J) ASCC3-depleted cells have a prolonged G1 arrest compared with control cells after anisomycin treatment. Error bars represent the SD. ∗∗p < 0.01. See also Figure S5.

Our data indicate that the ASCC cannot process 4NQO- or UVB-stalled ribosome complexes. Therefore, we investigated the physiological impact of unresolved stalled ribosomes on the cell. Ribosome stalling has been linked with stress-activated protein kinase (SAPK) signaling (Vind et al., 2020; Wu et al., 2020). The phosphorylation status of the SAPKs, p38^MAPK^ and JNK, was monitored in response to anisomycin and UVB. Although there was a transient increase in JNK phosphorylation after anisomycin, there was no effect on JNK signaling immediately after UVB (Figure 6A). In contrast, p38^MAPK^ signaling increased rapidly in response to both anisomycin and UVB, and the kinetics of p38^MAPK^ phosphorylation correlated with ribosome collisions in the cell (Figure 6A). Thus, UV-induced ASCC-resistant stalled ribosomes could provoke sustained p38^MAPK^ activation, whereas the resolution of anisomycin-stalled ribosomes could lead to transient p38^MAPK^ signaling. If this hypothesis is correct, inhibiting the resolution of anisomycin-stalled ribosomes should affect the kinetics of p38^MAPK^ signaling. We found that ASCC3 depletion prevented the resolution of anisomycin-stalled ribosomes for 6 h, and p38^MAPK^ phosphorylation was prolonged in response to these stalled ribosomes (Figures 6B and 6C). In comparison, ASCC3 depletion had little effect on p38^MAPK^ phosphorylation in cells exposed to UVB (Figure S5A). Sustained p38^MAPK^ signaling was also observed in ASC-1-depleted cells treated with anisomycin (Figure S5B). Thus, p38^MAPK^ signaling is stimulated by unresolved stalled ribosomes.

ZAK is known to activate the SAPK pathways in response to ribosome stalling (Vind et al., 2020; Wu et al., 2020). Depletion of ZAK inhibited p38^MAPK^ phosphorylation after UVB stress and after ASCC3 depleted cells were exposed to anisomycin (Figures 6D and 6E). Therefore, unresolved stalled ribosomes stimulate sustained ZAK-dependent p38^MAPK^ signaling.

The p38^MAPK^ pathway can promote cell death in certain contexts (Cuadrado and Nebreda, 2010). It has been proposed that activation of SAPKs by ZAK induces apoptosis when the cellular capacity to resolve stalled ribosomes is exceeded (Wu et al., 2020). However, we found that anisomycin treatment had no effect on cell death in either control or ASCC3-depleted cells regardless of the p53 status of the cell (Figures S5C and S5D). Therefore, unresolved stalled ribosomes do not promote cell death under these conditions.

Signaling through p38^MAPK^ can also activate adaptive cell survival mechanisms. In particular, p38^MAPK^ triggers cell-cycle arrest, facilitating adaptation to stress (Duch et al., 2012). Anisomycin treatment of asynchronous HeLa cells caused an increase in cells with 4N DNA content and a corresponding decrease in those with 2N DNA content (Figures 6F and S5E). This accumulation of 4N cells was more prominent in ASCC3-depleted cells, suggesting that unresolved stalled ribosomes cause a delay in cell-cycle progression at either G2 or M phase (Figures 6F and S5E). Since p38^MAPK^ is known to activate the G2/M cell-cycle checkpoint in response to DNA damage (Bulavin et al., 2001; Manke et al., 2005), we performed a mitotic trap assay to determine whether ribosome stalling can delay G2 to M-phase progression. HeLa cells were first treated with anisomycin to provoke ribosome stalling, after which they were exposed to nocodazole to trap the cells that transit through G2 into mitosis (Figure 6G). In the absence of anisomycin, cells accumulated the mitosis marker, phospho-histone H3, after nocodazole treatment (Figure 6G). After anisomycin treatment, there was a reduction in cells progressing into mitosis, which was more pronounced in ASCC3-depleted cells (Figure 6G). Therefore, anisomycin provokes a G2 arrest, which is prolonged when stalled ribosomes persist. Chemical inhibition of p38^MAPK^ (Figures 6H and S5F) or depletion of ZAK (Figures 6I and S5G) prevented the accumulation of cells in G2 after anisomycin treatment. Thus, unresolved stalled ribosomes activate ZAK and p38^MAPK^, triggering a delay in G2 phase.

The effect of ribosome stalling on G1-to-S-phase progression was determined using a mitotic trap assay. After adding nocodazole, control or ASCC3-depleted cells had almost exclusively 4N DNA content, showing that they were trapped in mitosis (Figures 6J and S5H). However, 12% of control siRNA-treated cells accumulated in G1 when they were exposed to anisomycin prior to nocodazole, suggesting that anisomycin caused a transient arrest in G1. After ASCC3 depletion, 36% of cells failed to progress from G1 upon anisomycin treatment (Figures 6J and S5H). Therefore, unresolved stalled ribosomes provoke a sustained G1 arrest.

It could be argued that these cell-cycle effects are caused by the inhibition of protein synthesis rather than ribosome stalling. However, the reduction in protein synthesis in response to anisomycin is not affected by ASCC3 depletion (Figure S5I), whereas the period of delay in G1 and G2 increases under the same conditions. Furthermore, interfering with ZAK-p38^MAPK^ signaling prevents G2 arrest, despite the continued presence of anisomycin (Figures 6H and 6I).

Our data support a model in which ribosome stalling delays progression through the cell cycle at G1 and G2. The duration of cell-cycle arrest depends on the number of unresolved stalled ribosomes. Hence, stalled ribosomes that are harder to resolve, such as ASCC-resistant stalls, will elicit a more protracted cell-cycle arrest.

## Discussion

Prior to nascent peptide extraction and degradation, a stalled ribosome must be split by the ASCC (Juszkiewicz et al., 2020; Matsuo et al., 2020). Our data confirm that the ASCC can resolve ribosomes stalled by anisomycin, emetine, mRNA methylation, aa-tRNA deficiency, and didemnin B (Figures 4A, 5F, S4F, and S4G). In contrast, the ribosome rescue factor Pelo cannot split internally stalled ribosomes *in vivo* (Figure S3B), in agreement with data showing that Pelo does not affect terminal stalling at a poly(A) site (Juszkiewicz et al., 2020). Since the ASCC can split ribosomes stalled in the pre-accommodation state with either an empty (MMS and aa-tRNA deficiency) or an occupied A-site (didemnin B), in the pre-peptide bond formation state (anisomycin), and in the pre-translocation state (emetine), the mechanism of resolution must be compatible with each of these structurally distinct stages of the elongation cycle. Furthermore, unlike Pelo, the ASCC can split ribosomes that have an occupied A-site because stalled ribosomes induced by emetine, anisomycin, or didemnin B can be resolved by the ASCC.

Of the four ASCC proteins, only ASCC3 and ASCC2 were known to play a role in RQC. ASCC3 helicase activity is essential to split the ribosome, and both ASCC3 and ASCC2 facilitate the stable arrest of the ribosome (Juszkiewicz et al., 2020; Matsuo et al., 2020). By measuring their effect on collided ribosomes, we show that ASC-1 and ASCC1 also contribute to the resolution of internally stalled ribosomes (Figure 4B). The loss of ASC-1 has a significant impact on ribosome splitting, implying an essential role for this factor in ribosome splitting, whereas the more modest effect of ASCC1 suggests that it may have an auxiliary role in the ASCC.

We found that UVB and 4NQO also stimulate ribosome stalling within the coding region of mRNA (Figure 2). This ribosome stalling is associated with a large increase in ASCC3 RNA binding directly linked to helicase activity (Figure 1), and an accumulation of the ASCC on the stalled ribosomes (Figure 2). Hence, the ASCC is recruited to these stalled ribosomes and the ASCC3 helicase engages with its RNA ligand. Given that RNA-helicase interactions usually occur with fast kinetics (Bohnsack et al., 2012; Hahn et al., 2012; Xiol et al., 2014), it was surprising to capture such a large increase in ASCC3 RNA binding. In addition, if the ASCC is recruited to stalled ribosomes in order to split them, how does this complex interact in a stable manner with polysomes? We found that UVB- and 4NQO-stalled ribosomes are long lived in the cell and that the ASCC plays no role in their delayed removal from the cell (Figures 3 and 4). In contrast, as TEI-stalled ribosomes are rapidly resolved by the ASCC, there is neither an accumulation of ASCC on TEI-stalled ribosomes nor a substantial increase in ASCC3 RNA binding in response to TEIs (Figure 3). Together, these data suggest that the ASCC is recruited to TEI-, UVB-, and 4NQO-stalled ribosomes, but it fails to resolve UVB- or 4NQO-stalled ribosomes, resulting in stable interaction of the ASCC with stalled ribosomes and of ASCC3 with its RNA ligand. Thus, we propose that resistance of a stalled ribosome to the ASCC helicase has a major influence on its longevity.

What is the nature of the damage inflicted by UVB or 4NQO that ultimately leads to ASCC-resistant stalled ribosomes? Ribosomes can eventually overcome the translation arrest imposed by 4NQO or UVB (Figures 5A and S4A), and the damage resulting in ASCC-resistant stalled ribosomes can occur in the absence of translating ribosomes (Figure 5B). Therefore, ASCC-resistant ribosome stalling cannot be a consequence of crosslinking of the ribosome, or another protein complex, to the mRNA. Neither can it be due to crosslinking of the ribosomal subunits or the aa-tRNA into the A-site. The appearance of UVB-induced ribosome collisions when ribosomes re-engage with the mRNA after a translation block is consistent with ribosome stalling due to damaged mRNA or aa-tRNA (Figure 5B). GCN2-dependent phosphorylation of eIF2α provides further support for a decoding issue after UVB or 4NQO stress (Figure 5C). We consider that damaged mRNA is more likely to be the cause of ASCC-resistant ribosome stalling for three reasons. First, highly structured RNA, such as tRNA, is far less sensitive to UV damage than single stranded RNA (Pearson and Johns, 1966). Second, ribosomes stalled by defective aa-tRNA that impairs codon recognition, accommodation, or the peptidyl transferase reaction would all be sensitive to ASCC (Figures 4A, S4F, and S4G). Third, in ribosomes stalled on defective mRNA, the damaged nucleobases would lie within the ribosome’s mRNA channel and could potentially interfere with ribosome splitting. Therefore, we propose that ASCC-resistant stalls are due to damaged mRNA.

UVB induces cyclobutane pyrimidine dimers between adjacent pyrimidines in RNA (Wurtmann and Wolin, 2009). We detected ribosome stalling on a pyrimidine tract using a UVB-irradiated mRNA (Figure 5D). Thus, dipyrimidine photoproducts in mRNA arrest the ribosome, which agrees with the observation that ribosomes accumulate at codons with adjacent pyrimidines after UVC (Wu et al., 2020). The structural limitations imposed by the cyclobutane ring between adjacent pyrimidines would undoubtedly hinder decoding. In yeast, ribosome stalling due to 4NQO has been attributed to ROS introducing 8-oxoguanosine into mRNA, thereby disrupting tRNA selection (Yan et al., 2019; Simms et al., 2014). However, we did not detect unresolved stalled ribosomes in HeLa cells exposed to β-lapachone, a redox cycling agent that generates ROS, or hydrogen peroxide (Figure S6), suggesting that 4NQO-induced ASCC-resistant ribosome stalling is not due to oxidative mRNA damage. 4NQO can also introduce an aminoquinoline 1-oxide adduct at the C8 and N2 positions of guanosine (Galiègue-Zouitina et al., 1985). In mammalian cells, these bulky RNA adducts could stall ribosomes by interfering with decoding. Given that ribosomes stalled by methylated nucleobases in the mRNA can be resolved by the ASCC (Figure 5F), we speculate that the structural constraints imposed by bulky or rotationally limiting mRNA adducts in the ribosome’s mRNA channel could create an energetic barrier to ribosome splitting by the ASCC helicase. In agreement with this hypothesis, MMC, which also introduces bulky adducts into nucleic acid, causes ASCC3 to accumulate on its RNA ligand (Figure 1). However, our data do not completely exclude the possibility that damaged aa-tRNA could associate with the ribosome in a manner that prevents splitting. Detailed structural studies will be required to determine precisely how specific RNA damage restricts ribosome splitting by the ASCC.

Recognition of unresolved stalled ribosomes by ZAK activates p38^MAPK^ and JNK (Vind et al., 2020; Wu et al., 2020). Long-lived unresolved stalled ribosomes arising from mRNA damage or defective RQC prolong this signaling, leading to a sustained arrest at the G2 phase of the cell cycle (Figure 6). Since ZAK depletion abrogates p38^MAPK^ phosphorylation, our data also suggest that ribosome stalling in the cytoplasm, and not nuclear DNA damage as was originally proposed (Manke et al., 2005), is the stimulus for p38^MAPK^ activation after UV stress (Figure 6D). Unresolved stalls also trigger a more protracted delay at the G1 phase of the cell cycle by an unknown mechanism (Figure 6J).

It has been suggested that ZAK stimulates cell death in response to excessive ribosome stalling (Wu et al., 2020). However, we found that unresolved stalled ribosomes had no effect on cell death (Figures S5C and S5D). Given that a much higher concentration of anisomycin was required to stimulate cell death than cell-cycle arrest, it is possible that ZAK can promote survival or death, tuning the cellular outcome to match the severity of ribosome stalling.

Overall, our data suggest that the mechanism and kinetics of stalled ribosome resolution are influenced by the nature of the stall. However, the cell initiates survival mechanisms in response to unresolved stalled ribosomes and can adapt accordingly.

### Limitations of the study

Although many types of stalled ribosomes are resolved by the ASCC, 4NQO- or UV-induced nucleobase damage results in ASCC-resistant stalled ribosomes. A high-resolution structural analysis of an ASCC-resistant stalled ribosome complex will highlight interactions between the bulky mRNA adducts and the ribosome that cause ribosome stalling. Complementary structural studies of a resistant stalled ribosome in complex with the ASCC will provide further mechanistic insight into the failure of ribosome splitting. Moreover, the identification of the ASCC3 RNA ligand in ASCC-resistant stalled ribosomes will facilitate an understanding of ASCC ribosome splitting. Finally, the mechanism for removing ASCC-resistant ribosomes from the cell requires further investigation.

## STAR★Methods

### Key resources table

**Table:** 

REAGENT or RESOURCE	SOURCE	IDENTIFIER
**Antibodies**		

Rabbit polyclonal anti-PABP	Abcam	Cat. #ab21060; RRID: AB_777008
Rabbit polyclonal anti-ASCC2	Abcam	Cat. #ab228827
Rabbit polyclonal anti-rpL11	Abcam	Cat. #ab79352; RRID: AB_2042832
Recombinant anti-phospho (S51) eIF2α	Abcam	Cat. #ab32157; RRID: AB_732117
Rabbit monoclonal anti-eIF4B	Abcam	Cat. #ab68474; RRID: AB_11156354
Rabbit polyclonal anti-ASC-1	Bethyl	Cat. #A300-203; RRID: AB_230268
Rabbit polyclonal anti-rpS3	Bethyl	Cat. #A303-840A; RRID: AB_2620191
Rabbit polyclonal anti-ZAK	Bethyl	Cat. #A301-993A; RRID: AB_1576612
Rabbit monoclonal anti-phospho (T180/Y182) p38^MAPK^	Cell Signaling	Cat. #4511; RRID: AB_2139682
Rabbit polyclonal anti-p38^MAPK^	Cell Signaling	Cat. #9212; RRID: AB_330713
Rabbit monoclonal anti-phospho (T183/Y185) JNK	Cell Signaling	Cat. #4671; RRID: AB_331338
Rabbit polyclonal anti-JNK	Cell Signaling	Cat. #9252; RRID: AB_2250373
Rabbit polyclonal anti-phospho (S10) histone H3	Cell Signaling	Cat. #9701; RRID: AB_331535
Rabbit monoclonal histone H3	Cell Signaling	Cat. #4499; RRID: AB_10544537
Rabbit polyclonal anti-PARP	Cell Signaling	Cat. #9542; RRID: AB_2160739
Rabbit polyclonal eIF2α	Cell Signaling	Cat. #9722; RRID: AB_2230924
Rabbit monoclonal anti-rpS6	Cell Signaling	Cat. #2217; RRID: AB_331355
Rabbit polyclonal anti-ZNF598	Novus Biologicals	Cat. #NBP1-84658; RRID: AB_11039996
Rabbit polyclonal anti-Pelo	Proteintech	Cat. #10582-1-AP; RRID: AB_2236833
Rabbit polyclonal anti-ASCC3	Proteintech	Cat. #17627-1-AP; RRID: AB_2059474
Rabbit polyclonal anti-ASCC1	Proteintech	Cat. #12301-1-AP; RRID: AB_2059350
Mouse monoclonal anti-FLAG	Sigma-Aldrich	Cat. #F1804; RRID: AB_262044
Rabbit polyclonal anti-tubulin	Thermo Fisher	Cat. #PA5-16863; RRID: AB_10986058
HRP conjugated goat anti-rabbit	GE Healthcare	Cat. #NA934V
HRP conjugated goat anti-mouse	DAKO	Cat. #P0447

**Bacterial and virus strains**		

XL10-Gold Ultracompetent Cells	Agilent Technologies	Cat. #200314
Biological samples		N/A

**Chemicals, peptides, and recombinant proteins**		

Hydrocortisone	Sigma-Aldrich	Cat. #H0888
Insulin	Sigma-Aldrich	Cat. #I9278
EGF	Peprotech	Cat. #AF-100-15
Cholera toxin	Sigma-Aldrich	Cat. #C8052
Zeocin	Thermo Fisher	Cat. #R25001
Blasticidin	Cambridge Bioscience	Cat. #B007-20ml
Hygromycin	Thermo Fisher	Cat. #10687010
Tetracycline	Sigma-Aldrich	Cat. #T7660
4-Nitroquinoline 1-oxide	Sigma-Aldrich	Cat. #8141
Mitomycin C	Sigma-Aldrich	Cat. #M0503
Doxorubicin	Sigma-Aldrich	Cat. #D1515
Camptothecin	Sigma-Aldrich	Cat. #C9911
Etoposide	Sigma-Aldrich	Cat. #E1383
Methyl methanesulfonate	Sigma-Aldrich	Cat. #129925
Puromycin	Sigma-Aldrich	Cat. #P8833
Harringtonine	Santa Cruz	Cat. #sc-204771
SB203580 (p38i)	Cell Signaling	Cat. #5633S
Nocodazole	Sigma-Aldrich	Cat. #SML1665
Anisomycin	Sigma-Aldrich	Cat. #A9789
Emetine	Santa Cruz	Cat. #sc-202600
Didemnin B	Prof. V. Ramakrishnan	N/A
Hippuristanol	Prof. Ya-Ching Chen	N/A
Cycloheximide	Sigma-Aldrich	Cat. #C7698
GCN2-IN-1 (GCN2i)	Cambridge Bioscience	Cat. #HY-10087
GSK2606414 (PERKi)	Sigma-Aldrich	Cat. #516535
Thapsigargin	Sigma-Aldrich	Cat. #T9033
β-lapachone	Sigma-Aldrich	Cat. #L2037
Complete EDTA-free protease inhibitor cocktail	Roche	Cat. #11836170001
Protease inhibitor cocktail	Sigma-Aldrich	Cat. #P8340
PhosSTOP phosphatase inhibitors	Roche	Cat. # 04906837001
NxGen RNase inhibitor	Lucigen	Cat. #30281
Micrococcal nuclease	New England Biolabs	Cat. #M0247
Turbonuclease	Sigma-Aldrich	Cat. #T4330
RNase A/T1	Thermo Fisher	Cat. #EN0551
Annexin V-FITC	Thermo Fisher	Cat. #A13199
DRAQ7	Thermo Fisher	Cat. #15106
Annexin binding buffer	BD Biosciences	Cat. #556454
FxCycle violet stain	Thermo Fisher	Cat. #F10347
L-[35S]-methionine (1]00 Ci/mmol)	Hartmann Analytic	Cat. #SCIS-103

**Critical commercial assays**		

ECL Prime Western Blotting Detection Reagent	GE Healthcare	Cat. #RPN2232
Q5 Site-directed Mutagenesis Kit	New England Biolabs	Cat. #E0554S
Vaccinia Capping System	New England Biolabs	Cat. #M2080S
TranscriptAid T7 High Yield Transcription Kit	Thermo Fisher	Cat. #K0441
Flexi® Rabbit Reticulocyte Lysate System	Promega	Cat. #L4540

**Deposited data**		

Mass spectrometry data from cytoplasmic RIC analysis of MCF10A cells – experiment 1	This paper	PRIDE: PXD023976
Mass spectrometry data from cytoplasmic RIC analysis of MCF10A cells – experiment 2	This paper	PRIDE: PXD023978
Mass spectrometry data from cytoplasmic RIC analysis of mock and UVB exposed MCF10A – experiment 1	This paper	PRIDE: PXD023861
Mass spectrometry data from cytoplasmic RIC analysis of mock and UVB exposed MCF10A – experiment 2	This paper	PRIDE: PXD023878

**Experimental models: Cell lines**		

MCF10A	ATCC	CRL-10317
HeLa	ATCC	CCL-2
293 Flp-In T-REx™	Thermo Fisher	R78007
293 Flp-In T-REx™ ASCC3-3F	This paper	N/A
293 Flp-In T-REx™ 3F-ASCC3	This paper	N/A
293 Flp-In T-REx™ ASCC3-F^AAA^	This paper	N/A
293 Flp-In T-REx™ ASCC3-F^E612Q^	This paper	N/A

**Oligonucleotides**		

See Table S3 for oligonucleotides, gblocks and siRNAs	This study	N/A

**Recombinant DNA**		

pcDNA5.1/FRT/TO	Thermo Fisher	Cat. #V652020
pcDNA5.1/FRT/TO/C3F	This paper	N/A
pcDNA5.1/FRT/TO/N3F	This paper	N/A
pcDNA5.1/FRT/TO/ASCC3-3F	This paper	N/A
pcDNA5.1/FRT/TO/3F-ASCC3	This paper	N/A
pcDNA5.1/FRT/TO/3F-ASCC3^AAA^	This paper	N/A
pcDNA5.1/FRT/TO/ASCC3^E612Q^-3F	This paper	N/A
pUC18	Thermo Fisher	Cat. #SD0051
pUCK100	This paper	N/A
pUCK100Fluc	This paper	N/A
pUCK100YY	This paper	N/A
pcDNA3.1Fluc	This paper	N/A

**Software and algorithms**		

FlowJo	FLowJo LLC	https://www.flowjo.com/
PeakTrak	Teledyne ISCO	https://www.teledyneisco.com/
GraphPad Prism	GraphPad Software	https://www.graphpad.com/

**Other**		

Fetal Bovine Serum	Sigma-Aldrich	Cat. #F9665
Horse Serum	Thermo Fisher	Cat. #16050122
Tetracycline-free Fetal Bovine Serum	Biosera	Cat. #FB-1001T
Lipofectamine RNAiMAX	Thermo Fisher	Cat. #13778150
Immun-Blot PVDF Membrane	Biorad	Cat. #1620177
ProtoSafe Blue Colloidal Coomassie Stain	National Diagnostics	Cat. #EC-722
BamHI-HF	New England Biolabs	Cat. #R3136
XhoI	New England Biolabs	Cat. #R0146
Acc65I	New England Biolabs	Cat. #R0599
ApaI	New England Biolabs	Cat. #R0114
XbaI	New England Biolabs	Cat. #R0145S
HindIII	New England Biolabs	Cat. #R0104S
NheI	New England Biolabs	Cat. # R3131S
T4 DNA Ligase	New England Biolabs	Cat. #M0202
Oligo(dT)_25_ Magnetic Beads	New England Biolabs	Cat. #S1419S
DMEM minus arginine and lysine	Thermo Fisher	Cat. #88364

### Resource availability

#### Lead contact

Further information and requests for resources and reagents should be directed to and will be fulfilled by the lead contact, Anne Willis (aew80@mrc-tox.cam.ac.uk)

#### Materials availability

All unique/stable reagents generated in this study are available from the lead contact without restriction.

### Experimental model and subject details

MCF10A cells were cultured in Dulbecco’s Modified Eagle medium/Ham’s F12 (DMEM/F12) supplemented with 5% horse serum, 0.5 mg/ml hydrocortisone, 10 μg/ml insulin, 20 ng/ml EGF and 20 ng/ml cholera toxin. HeLa cells were cultured in DMEM containing 10% fetal bovine serum. Parental HEK293 Flp-In T-REx cells were cultured in DMEM with 10% tetracycline-free fetal bovine serum, 100 μg/ml Zeocin and 15 μg/ml blasticidin. Stable cell lines containing tetracycline-inducible FLAG-tagged ASCC3 variants were generated according to the manufacturer’s guidelines (Thermo Fisher) and cultured in DMEM with 10% tetracycline-free fetal bovine serum, 15 μg/ml blasticidin and 150 μg/ml hygromycin B. Expression of the FLAG-tagged ASCC3 variants was induced for 20 hours using 1 μg/ml tetracycline. Gene silencing using siRNAs was performed for 3 days using Lipofectamine RNAiMAX according to the manufacturer’s recommendations. Cells were treated with 20 μM 4NQO, 20 μM mitomycin C, 1 μM doxorubicin, 20 μM camptothecin, 20 μM etoposide, 100 μg/ml puromycin, 1 μM hippuristanol, 2 μg/ml harringtonine, 0.25 mg/ml MMS, 10 μM SB203580 (p38i), 5 μM GCN2-IN-1 (GCN2i), 1 μM GSK2606414 (PERKi), 0.5 μM thapsigargin, 20 μM β-lapachone or 0.2 μM didemnin B unless otherwise stated. Before exposing cells to UVB, the media was removed, and the cells were washed twice with PBS. The cells were then exposed to 300 J/m^2^ UVB (302 nm), unless otherwise stated, using a CL-1000M crosslinker (UVP, 95-0230-02) and the media was replaced. Cells were exposed to UVC (254 nM) using a CL-1000S crosslinker (UVP, 95-0174-01). In the amino acid depletion experiments, cells were grown in DMEM minus arginine and lysine supplemented with dialysed fetal bovine serum, and where specified GCN2-IN-1.

### Method details

#### Constructs and antibodies

A C-terminal 3XFLAG sequence was inserted into pcDNA5/FRT/TO using the C-3XFLAG gblock between the *Xho*I and *Apa*I sites to create pcDNA5/FRT/TO/C3F. The ASCC3 coding sequence was amplified from a cDNA clone (Source Bioscience, OCAAo5051B065D) and inserted between the *Acc65*I and *Xho*I sites of pcDNA5/FRT/TO/C3F to create pcDNA5/FRT/TO/ASCC3-3F, the C-terminally FLAG-tagged ASCC3 inducible expression construct. An N-terminal 3XFLAG sequence was inserted into pcDNA5/FRT/TO using the N-3XFLAG gblock between the *Bam*HI and *Acc65*I sites to create pcDNA5/FRT/TO/N3F. The ASCC3 coding sequence was inserted between the *Bam*HI and *Xho*I sites of pcDNA5/FRT/TO/N3F to create pcDNA5/FRT/TO/3F-ASCC3, the N-terminally FLAG-tagged ASCC3 inducible expression construct. The GKT sequence of ASCC3 (residues 504-506) in pcDNA5/FRT/TO/3F-ASCC3 was altered to AAA using site-directed mutagenesis to create the ASCC3-F^AAA^ mutant construct. Glutamic acid 612 of ASCC3 in pcDNA5/FRT/TO/ASCC3-3F was changed to glutamine to create the ASCC3^E612Q^ mutant construct. Site-directed mutagenesis was performed using a Q5 site-directed mutagenesis kit. To construct pUCK100, a segment incorporating the T7 promoter sequence, multiple cloning site, and 80 nucleotide poly(A) sequence was amplified by overlap extension PCR using T7_5prime_ultramer_F, XbaI_polyA_ultramer_R, T7_5prime_F, and NcoI_Fluc_F. The PCR product was inserted into pUC18 between the *Hind*III and *Xba*I sites. A unique *Nhe*I site was inserted into the resulting construct by overlap extension PCR using NheI_Fluc_F, NheI_Fluc_R, M13/pUC_F, and M13/pUC_R to produce pUCK100. The Firefly luciferase coding region was amplified from pcDNA3.1Fluc using NcoI_Fluc_F and XhoI_Fluc_R and inserted into pUCK100 using *Nco*I and *Xho*I to produce pUCK100Fluc. The UVPD reporter coding sequence contains 611 nucleotides with no adjacent pyrimidines (noYY), followed by a pyrimidine-rich tract (YY, CCACTTCCTCTTCCCTTCTCCCCTC) and 240 nucleotides of additional coding sequence. To create the UVPD reporter construct (pUCK100YY), a custom plasmid encoding the UVPD reporter coding region was synthesised by Genscript Biotech (Piscataway, USA), from which the UVPD sequence was excised and inserted into pUCK100 using *Nco*I and *Nhe*I. Plasmid sequences were confirmed by Sanger sequencing.

#### Western blot analysis

For the analysis of cytoplasmic proteins, cells were washed with PBS prior to lysis with cytoplasmic lysis buffer (20 mM Hepes pH 7.5, 10 mM NaCl, 5 mM MgCl_2_, 0.2 M sucrose, 0.5% NP40) supplemented with Complete protease inhibitors. PhosSTOP protein phosphatase inhibitors were included in the lysis buffer to analyse protein phosphorylation. Total protein was extracted using 20 mM Hepes pH 7.5, 150 mM NaCl, 1% NP40, 0.5% deoxycholate, 0.1% SDS, 1 mM MgCl_2_, 1X Complete protease inhibitors, followed by digestion of the chromatin with 125U Turbonuclease for one hour on ice and finally the lysate was supplemented with 1% SDS and heated at 70^°^C for 5 minutes. Proteins were extracted from nuclear pellets using the same method. Typically, 20 μg of protein was analysed for each sample. For sucrose density gradient fractions approximately 3% of each fraction was analysed. Samples were resolved on 4-12% NuPAGE gels and transferred to PVDF membrane. Membranes were blocked in 5% non-fat skimmed milk in TBST (20 mM Tris pH 8, 150 mM NaCl, 0.1% Tween-20) for one hour at room temperature. Primary antibodies were diluted in 5% non-fat skimmed milk in TBST or in the case of phospho-specific antibodies in 5% BSA in TBST. Primary antibodies were detected using HRP-conjugated secondary antibodies and ECL Prime reagents. All antibodies used in this study are listed in the key resources table.

#### Cytoplasmic-RNA interactome capture (RIC)

For cytoplasmic-RIC combined with western analysis, approximately 1-2 x 10^7^ control and treated cells were crosslinked using 150 mJ/cm^2^ UVC. Cells were harvested by scraping into cytoplasmic lysis buffer supplemented with 1X Complete EDTA-free protease inhibitors and 200U NxGen RNase inhibitor. After the nuclei were pelleted by centrifugation at 2,000 x g for 5 minutes at 4^°^C, lithium chloride (LiCl), lithium dodecyl sulphate (LiDS), and DTT were added to the cytoplasmic lysate to a final concentration of 0.5 M, 0.75% and 5 mM, respectively. Lysates were incubated with oligo(dT)_25_ beads for one hour at room temperature with mixing. The beads were washed for 5 minutes with 20 mM Tris pH 7.4, 0.5 M LiCl, 0.5% LiDS, 5 mM DTT and then washed twice for 5 minutes with 20 mM Tris pH 7.4, 0.5 M LiCl, 0.1% LiDS, 5 mM DTT. Subsequently, the beads were washed twice with 20 mM Tris pH 7.4, 0.1 M LiCl, 0.1% LiDS, 5 mM DTT and 20 mM Tris pH 7.4, 0.1 M LiCl. RNA was eluted after resuspending the beads in 20 mM Tris pH 7.4 and incubating the beads at 70^°^C for 3 minutes. RNA was digested with 40 μg/ml RNase A, 100U/ml RNase T1 and 125U Turbonuclease for 2 hours. RIC samples were subjected to western analysis to investigate specific RNA-protein interactions. RIC samples were normalized according to RNA content with typically 5-20 μg of RNA loaded per sample. In addition, 0.5-1% of each input lysate was loaded.

#### Quantitative mass spectrometry

LC–MS/MS was used to identify and quantify RNA binding proteins (RBPs). UV crosslinked RBPs isolated from 2 x 10^8^ MCF10A cells were separated by SDS PAGE and serial gel slices were digested *in situ* with trypsin (Dickens et al., 2012). Extracted tryptic peptides were analysed using data-independent acquisition (DIA) on a nanoAcquity UPLC system coupled to a Waters Synapt G2-S HDMS mass spectrometer. The ISOQUANT ‘TOP 3’ method was used for quantification of proteins (Kuharev et al., 2015). Two independent biological repeats of each experiment were analysed. Only those proteins found in both repeats were included in the analysis. In the comparison of control and UVB-treated RNA-protein interactions, RNA binding proteins with missing values in one of the conditions were excluded from the analysis.

#### Sucrose density gradient centrifugation

Lysates were layered on to 10-50% sucrose gradients prepared in 20 mM Hepes pH 7.5, 100 mM NaCl, 5 mM MgCl_2_ and 100 μg/ml cycloheximide. Gradients were subjected to centrifugation at 38,000 rpm in a Sorvall TH64.1 rotor for 2 or 2.5 hours at 4^°^C. Fractions were collected from the top of the gradient using an ISCO density gradient fractionation system with continuous monitoring of the absorbance at 254 nm.

#### Analysis of ASCC protein association with polyribosomes

Prior to harvesting, cells were treated with 100 μg/ml cycloheximide for three minutes and harvested using trypsin. Cells were swollen in 20 mM Hepes pH 7.5, 10 mM NaCl, 3 mM MgCl_2_, 100 μg/ml cycloheximide, 1X protease inhibitor cocktail, 200U NxGen RNase inhibitor for 20 minutes and then passed repeatedly through a 27G needle to achieve lysis. After lysates were clarified by centrifugation, the NaCl was adjusted to 100 mM and the lysates were resolved on sucrose gradients. Following fractionation, samples of the fractions were precipitated using chloroform-methanol. Precipitated proteins were resuspended in 2X NuPAGE sample buffer (Thermo Fisher) and proteins were detected by western analysis. Polysomes were dissociated by adding 20 mM EDTA to the cell lysate. Cells were treated with 100 μg/ml puromycin to block translation prior to sucrose density gradient analysis. Cell lysates were incubated with 1000U micrococcal nuclease for 30 minutes at 22^°^C to digest polysomes.

For *in vivo* crosslinking prior to sucrose density gradient analysis, adherent cells were treated with 0.2% formaldehyde in PBS for 5 minutes. Subsequently, the formaldehyde was quenched with 100 mM Tris pH 7.4. Lysates were prepared by scraping the cells into 20 mM Hepes pH 7.5, 100 mM NaCl, 5 mM MgCl_2_, 1% Triton X-100, 0.5% sodium deoxycholate, 1X protease inhibitor cocktail, 200U NxGen RNase inhibitor and 100 μg/ml cycloheximide. Clarified lysates were resolved using sucrose density gradient analysis and fractions were processed as above.

#### Harringtonine run-off assays

Harringtonine blocks the 80S ribosome at the initiation codon and prevents further initiating ribosomes from progressing into translation elongation (Ingolia et al., 2011). Ribosomes run off the mRNA and the rate of polysome loss is measured using sucrose density gradient centrifugation (Knight et al., 2015).

HeLa cells were treated with 20 μM 4NQO or 300 J/m^2^ UVB for one hour. Translation was blocked by treating the cells with 2 μg/ml harringtonine for 0, 120 or 180 seconds. After which, the cells were incubated with 100 μg/ml cycloheximide for 3 minutes. Lysates were prepared by scraping the cells into 20 mM Hepes pH 7.5, 150 mM NaCl, 3 mM MgCl_2_, 0.2 M sucrose, 0.5% NP40, 100 μg/ml cycloheximide, 1X Complete protease inhibitor cocktail, 200U NxGen RNase inhibitor. An equivalent amount of protein from each extract was resolved on 10-50% sucrose gradients.

#### Ribosome collision assays

After cells were exposed to various treatments, cytosolic lysates were prepared using 20 mM Hepes pH 7.5, 100 mM NaCl, 5 mM MgCl_2_, 100 μg/ml digitonin, 100 μg/ml cycloheximide, 1X protease inhibitor cocktail, 200U NxGen RNase inhibitor. Extracts were incubated on ice for 5 minutes prior to centrifugation at 17,000 x g for 5 minutes at 4^°^C. After adding calcium chloride to a final concentration of 1 mM, lysates were digested with 500U micrococcal nuclease for 30 minutes at 22^°^C. Digestion was terminated by adding 2 mM EGTA. Equivalent amounts of lysate (2-2.5 mg) were resolved on 10-50% sucrose gradients for two hours. The gradients were passed through an ISCO density gradient fractionation system with continuous monitoring of the absorbance at 254 nm. Three independent biological repeats of each ribosome collision assay were performed. A representative assay is shown for each experiment.

For the kinetic analysis and RQC factor depletion experiments, cells were exposed to 0.2 μM emetine and 20 μM 4NQO for 15 minutes, respectively, after which the cells were washed twice with PBS and allowed to recover in fresh media. Given that the binding of emetine to the ribosome is effectively irreversible (Grollman, 1968), emetine will remain bound to the ribosome when it is removed from the media in these experiments. Anisomycin binds less tightly to the ribosome (Barbacid and Vazquez, 1974) and therefore we used a continuous treatment of 0.2 μM anisomycin to assay the accumulation and subsequent resolution of stalled ribosomes. Cells were exposed to 0.2 μM didemnin B (a kind gift from Prof. V. Ramakrishnan) for 15 minutes. After which, the cells were allowed to recover in fresh media for 1 hour. Cells were exposed to UVB as previously described.

For the harringtonine run-off experiments (Figures 5A and S4A), the cells were exposed to 20 μM 4NQO for 15 minutes or 600 J/m^2^ UVB and then to 2 μg/ml harringtonine for the specified times. After which, ribosome collision assays were performed.

In order to damage RNA when ribosomes are not associate with mRNA (Figures 5B and S4B–S4D), HeLa cells were first exposed to 1 μM hippuristanol for 30 minutes. After which the media was removed, and the cells were washed extensively with PBS. Cells were irradiated with 600 J/m^2^ UVB and allowed to recover in fresh media.

#### mRNA synthesis and *in vitro* translation

pUCK100Fluc and pUCK100YY were digested with *Xba*I and purified to produce template DNA. Fluc mRNA and UVPD reporter mRNAs were produced by *in vitro* transcription of template DNAs using TranscriptAid T7 High Yield Transcription Kit. Transcripts were 5’-capped using a Vaccinia Capping System and purified by phenol/chloroform extraction. Transcripts were quantified using their absorbance at 254 nM and analysed by denaturing agarose gel electrophoresis to confirm their size and integrity. Fluc mRNA or UVPD mRNAs were exposed to UVB radiation at room temperature using a CL-1000M crosslinker (UVP, 95-0230-02). Fluc RNA was exposed to 1200 J/m^2^ UVB. In order to detect the noYY nascent chain-tRNA, the RNA was irradiated with 1200, 2400 and 4800 J/m^2^ UVB. The mRNAs were translated for 45 minutes using a Flexi® Rabbit Reticulocyte Lysate System in the presence of L-[35S]-methionine. Reactions were treated with Benzonase nuclease and RNase A/T1 for 10 minutes, where specified, and stopped by addition of SDS-PAGE buffer. Reactions were resolved by SDS-PAGE and dried gels were exposed to X-ray film to obtain autoradiographs.

#### Flow cytometry

All flow cytometry data were acquired using a BD LSRFortessa (BD Biosciences). A total of 10,000 counts were acquired for each experimental condition, and all flow cytometry data were analysed with FlowJo data analysis software (version 10.1). Three independent experiments were performed and analysed in each case.

Cell death was quantified by measuring annexin V–fluorescein isothiocyanate (FITC) binding to externalized phosphatidylserine with a 488 nM laser and DRAQ7 uptake in the cell with a 561 nM laser. Cells were collected and washed in PBS before resuspension in annexin binding buffer. Cells were incubated with annexin V-FITC and DRAQ7 for 20 min before analysis. Cell death was determined at 16 hours after treatment with 0.2 μM anisomycin.

FxCycle violet stain (4′, 6-diamidino-2-phenylindole, dihydrochloride) was used to determine the cell cycle distribution of a population of cells. After drug treatment, cells were collected and fixed in ice-cold 70% ethanol. To quantify the DNA content of the cells, FxCycle violet stain was incubated with the cells for 16 hours at 4°C and subsequently the binding of the dye to DNA was detected with a 405 nM laser. Cell cycle state was determined using Watson’s (pragmatic) model. The effect of anisomycin on cell cycle distribution in an asynchronous population of cells was determined after 16 hours treatment with 0.2 μM anisomycin. Cells were pre-treated with 10 μM SB203580 for one hour to block p38^MAPK^ signaling.

Nocodazole prevents cells from transiting from M-phase into the next G1-phase, allowing the study of cells progressing out of G1 phase. To determine the effect of anisomycin on the G1 phase (Figure 6J), cells were first treated with 0.2 μM anisomycin for 6 hours and then with 50 ng/ml nocodazole for 16 hours.

#### *In vivo*^35^S-methionine/cysteine incorporation

Cells were labelled with ^35^S-methionine/cysteine (Met/Cys) for 30 minutes at 37 °C in a 5% CO_2_ incubator. Radiolabelled Met/Cys incorporation was stopped by the addition of chilled PBS. Cells were lysed in radioimmunoprecipitation assay buffer (50 mM Tris-HCl pH 7.5, 150 mM NaCl, 1% NP-40, 0.5% sodium deoxycholate, 0.1% sodium docecyl sulphate, 1 mM EDTA), and proteins were precipitated with trichloroacetic acid. Met/Cys incorporation into newly synthesised proteins was evaluated by scintillation counting. A bicinchoninic acid assay was performed on the sample extracts to normalise the protein concentration.

### Quantification and statistical analysis

Western analysis was performed in three independent experiments and blots were quantified by densitometry using Fiji software. Most statistical analyses for significance used an unpaired Student’s t-test. ^∗^*P* < 0.05, ^∗∗^*P* < 0.01, ^∗∗∗^*P* < 0.001 were considered to be statistically significant. In Figures S5C, S5D, and S5I, a two-way ANOVA with Tukey’s multiple comparison test was performed to compare each condition. Error bars are the standard deviation of the mean in three independent experiments.

## Data Availability

• Mass spectrometry data have been deposited to the PRIDE database and are publicly available as of the date of publication. Accession numbers are listed in the key resources table. • This paper does not report original code. • Any additional information required to reanalyze the data reported in this paper is available from the lead contact upon request.

## Supplemental information

Document S1. Figures S1–S6

Table S1. Mass spectrometry analysis of proteins isolated by RIC to identify candidate cytoplasmic RBPs, related to Figure 1

Table S2. Mass spectrometry analysis of proteins isolated by cytoplasmic RIC to identify RBPs with altered RNA-protein interaction after UVB stress, related to Figure 1

Table S3. Oligonucleotides, gblocks, and siRNAs used in this study, related to STAR Methods

Document S2. Article plus supplemental information
