# Supplementary material for: Unresolved stalled ribosome complexes restrict cell-cycle progression after genotoxic stress
Source: Mol Cell. 2022 Apr 21;82(8):1557–1572.e7. doi: 10.1016/j.molcel.2022.01.019 (PMC9098122; doi:10.1016/j.molcel.2022.01.019)
Supplement: Document S1. Figures S1–S6 [file mmc1.pdf]

Molecular Cell, Volume *82*

Supplemental information

Unresolved stalled ribosome complexes restrict cell-cycle progression after genotoxic stress

Mark Stoneley, Robert F. Harvey, Thomas E. Mulroney, Ryan Mordue, Rebekah Jukes-Jones, Kelvin Cain, Kathryn S. Lilley, Ritwick Sawarkar, and Anne E. Willis

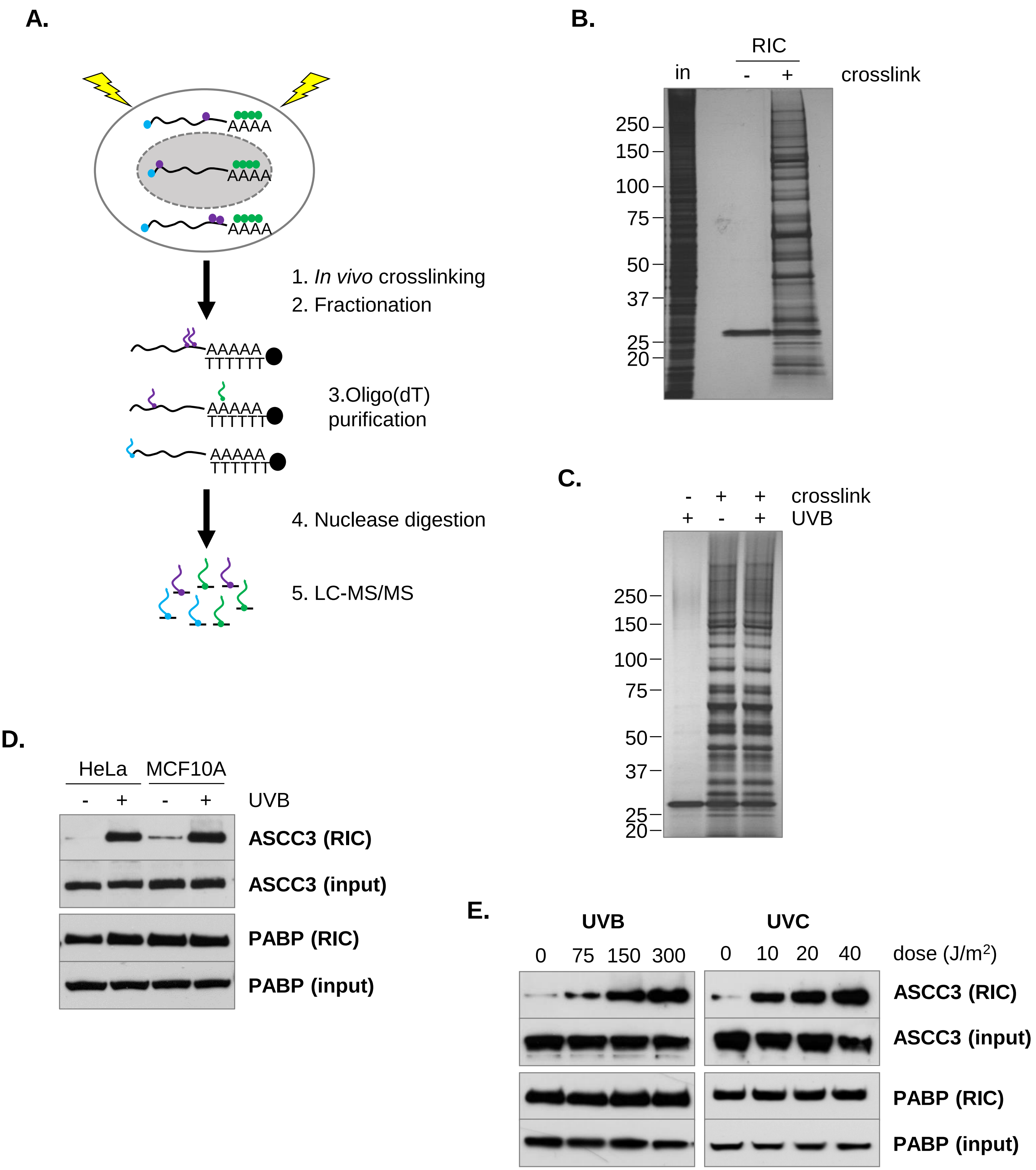

Figure S1. Bulky nucleic acid damage induces ASCC3 RNA binding. Related to Figure 1.
(A) Schematic detailing the use of fractionation combined with RNA interactome capture (RIC) to identify RBPs in a subcellular compartment. Cells are exposed to UVC light to crosslink RNA and proteins *in vivo* (1). Cell fractionation is performed (2) and the RNA and covalently linked RBPs are isolated from the chosen fraction using oligo(dT) beads (3). The RNA is digested with nucleases (4) leaving RBPs that can be identified by mass spectrometry (5). **(B)** Proteins eluted from a RIC experiment performed on untreated MCF10A cells (lane 3) or cells crosslinked with 150 mJ/cm^2 UVC (lane 4). The input lysate (in) is shown for comparison. **(C)** RIC eluates from MCF10A cells treated with 300 J/m^2 UVB (lane 3) and mock-treated cells (lane 2). Few proteins are recovered from cells irradiated with 300 J/m^2 UVB in the absence of UVC crosslinking (lane 1), showing that this dose of UVB does not have sufficient energy to crosslink proteins to RNA. **(D)** RIC experiment showing that UVB irradiation stimulates comparable levels of ASCC3 RNA binding in HeLa and MCF10A cells. **(E)** ASCC3 RNA binding increases in a dose-dependent manner after UVB and UVC irradiation of MCF10A cells.

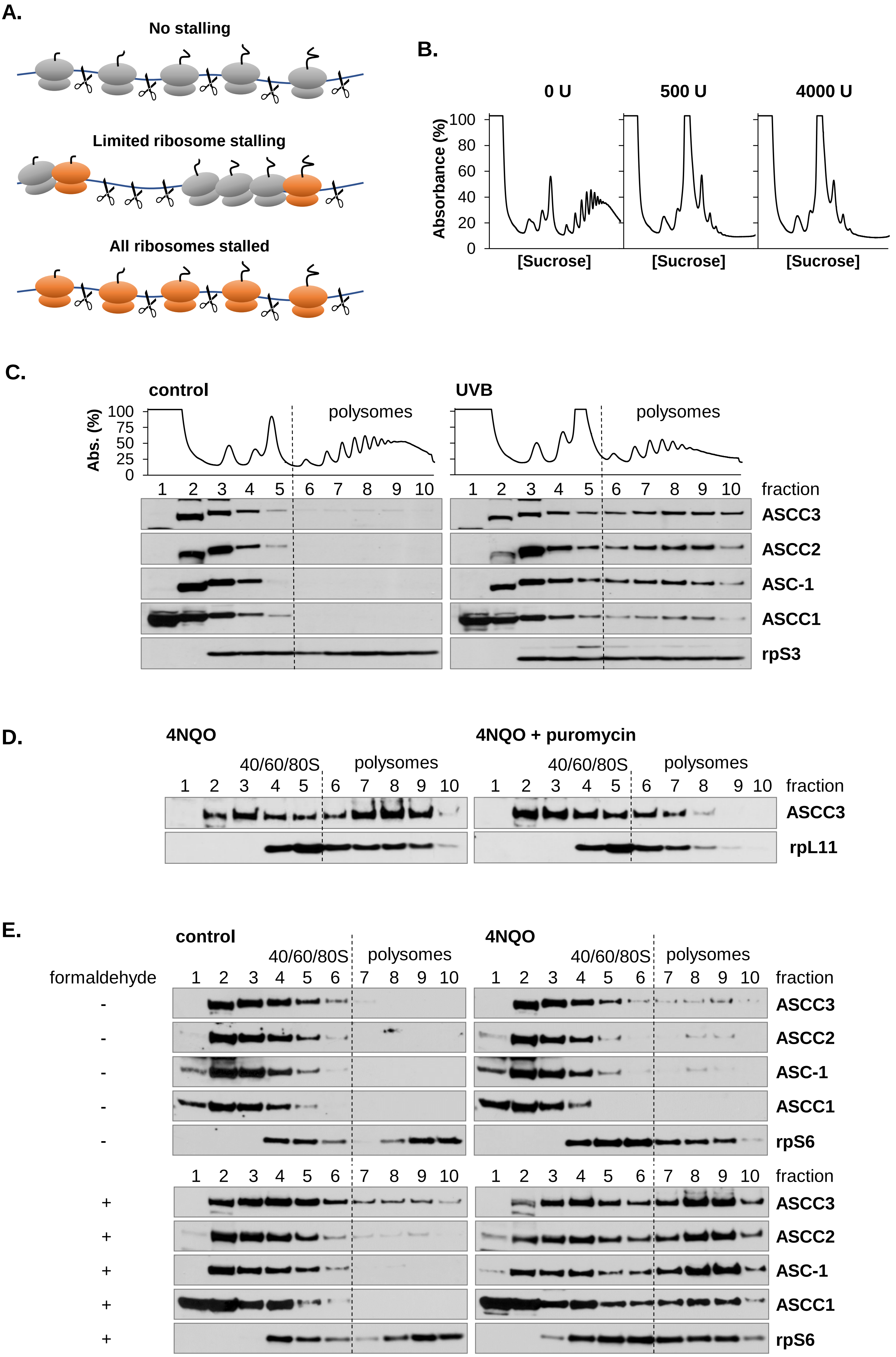
A.
No stalling
Limited ribosome stalling
All ribosomes stalled
B.
0 U
500 U
4000 U
Absorbance (%)
[Sucrose]
C.
control
UVB
polysomes
Abs. (%)
fraction
ASCC3
ASCC2
ASC-1
ASCC1
rpS3
D.
4NQO
4NQO + puromycin
40/60/80S
polysomes
fraction
ASCC3
rpL11
E.
control
4NQO
40/60/80S
polysomes
formaldehyde
fraction
ASCC3
ASCC2
ASC-1
ASCC1
rpS6

Figure S2. The ASCC accumulates on UVB and 4NQO-stalled ribosomes. Related to Figure 2.

(A) Micrococcal nuclease (MNase) digests the mRNA of a polyribosome in the inter-ribosomal region producing mainly 80S ribosomes. Limited ribosome stalling on an mRNA will cause ribosome collisions that protect regions of the mRNA from MNase digestion. Thus, ribosome collisions can be detected due to the release of MNase-resistant polysomes after digestion. If all of the ribosomes on a polysome are stalled there will be no collisions and MNase digestion will result in the release of mainly 80S ribosomes. **(B)** Sucrose density gradient analysis of HeLa cytoplasmic lysates treated with 500U or 4000U of MNase. **(C)** The distribution of ASCC3, ASCC2, ASC-1 and ASCC1 after sucrose density gradient centrifugation of control HeLa cell lysate (left) and lysate from cells treated with 300 J/m^2 UVB (right). **(D)** Inhibition of translation with puromycin prevents ASCC3 from accumulating on polysomes after 4NQO stress. **(E)** Control HeLa cells (left) or cells treated with 20 μM 4NQO (right) were lysed one hour after treatment (top panel) or were subjected to limited *in vivo* formaldehyde crosslinking prior to cell lysis (bottom panel). After sucrose density gradient centrifugation of the lysates, the distribution of the ASCC proteins was monitored by western analysis. Accumulation of the ASCC proteins on the polysomes after 4NQO treatment can be seen only after *in vivo* crosslinking (lower panel) because the lysis buffer conditions disrupt the interaction between the ASCC and the polysomes in the absence of crosslinking (upper panel).

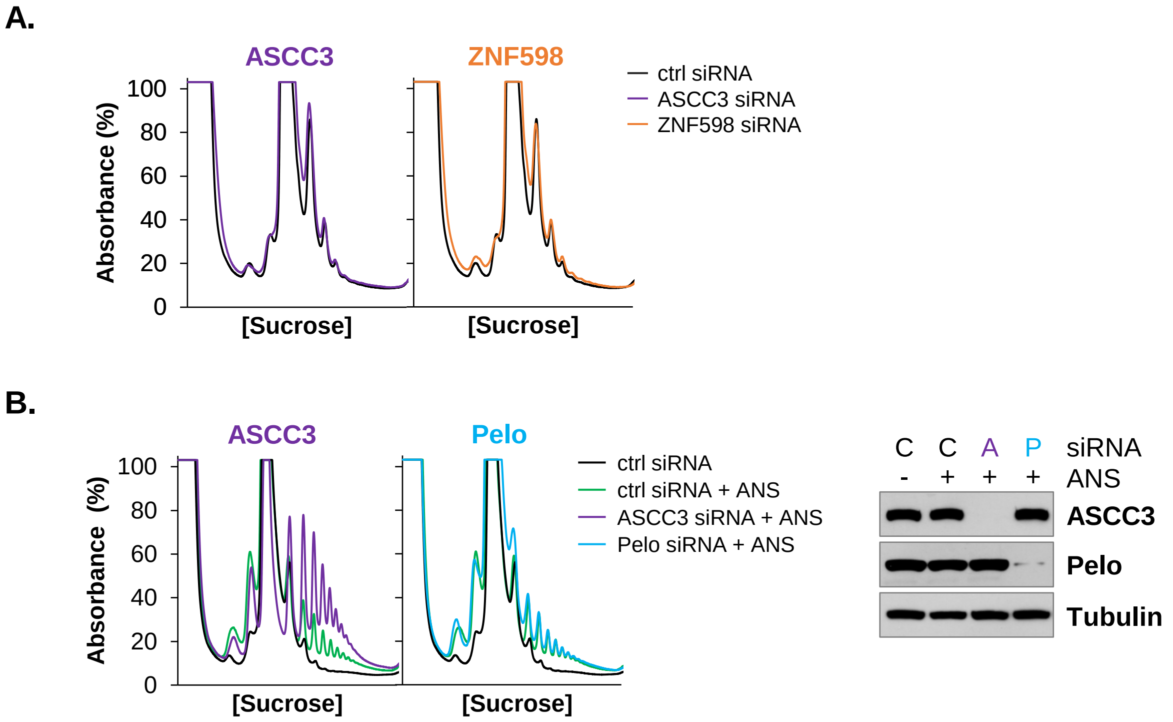

Figure S3. UVB or 4NQO-stalled ribosomes are resistant to the ASCC. Related to Figure 4.
(**A)** The effect of ASCC3 or ZNF598 depletion on the number of collided ribosomes in untreated HeLa cells. Depletion of these proteins consistently had no effect on MNase resistant-polysomes in these experiments. **(B)** The effect of ASCC3 or Pelo depletion on ribosome collisions one hour after treatment of HeLa cells with 0.2 μM anisomycin. ASCC3 (A) and Pelo (P) siRNAs efficiently depleted the corresponding proteins compared to the control siRNA (C) in these experiments.

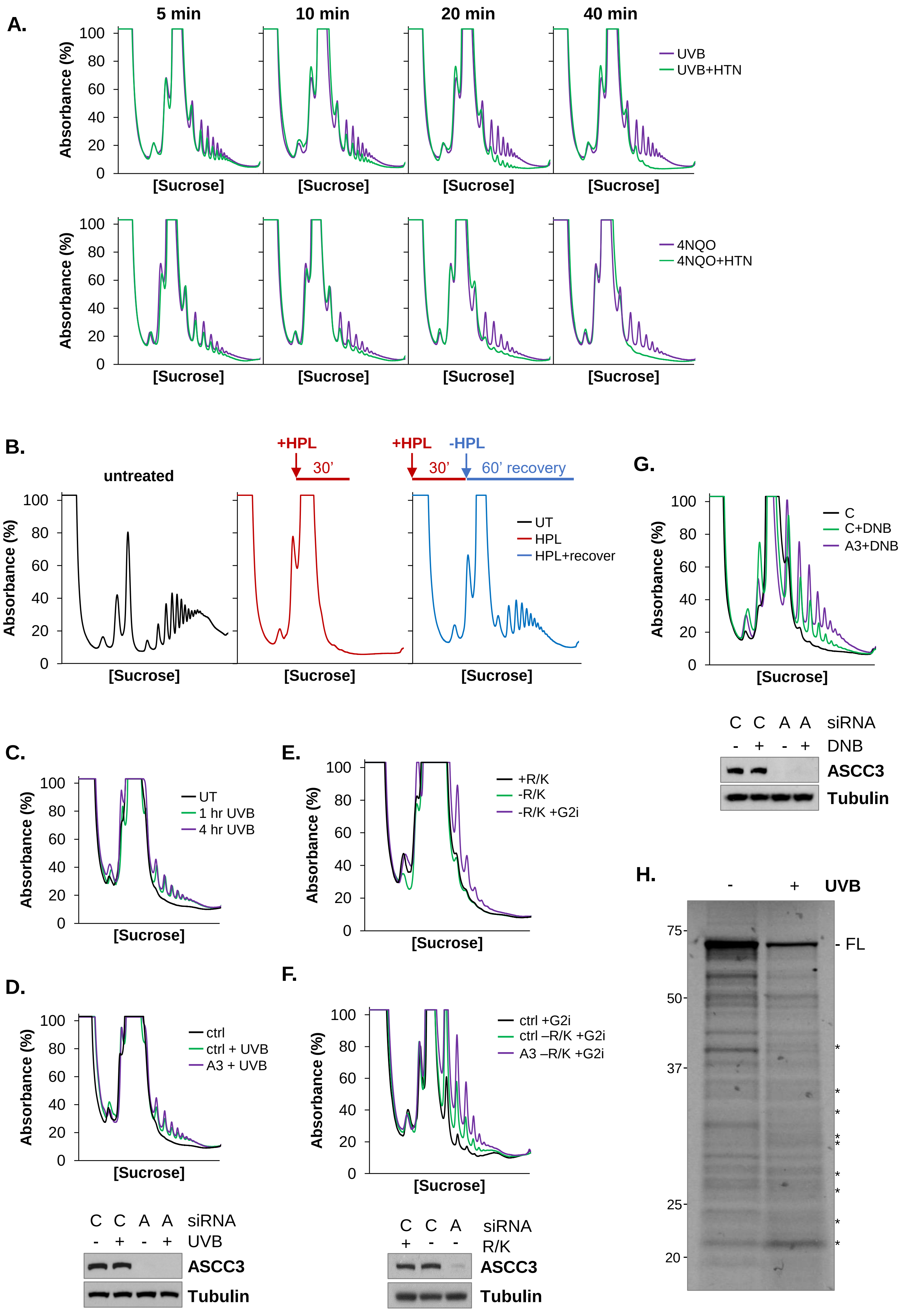
A.
5 min
10 min
20 min
40 min
Absorbance (%)
[Sucrose]
UVB
UVB+HTN
4NQO
4NQO+HTN
B.
untreated
+HPL
30’
-HPL
60’ recovery
UT
HPL
HPL+recover
C.
UT
1 hr UVB
4 hr UVB
D.
ctrl
ctrl + UVB
A3 + UVB
C C A A siRNA
- + - + UVB
ASCC3
Tubulin
E.
+R/K
-R/K
-R/K +G2i
F.
ctrl +G2i
ctrl –R/K +G2i
A3 –R/K +G2i
C C A siRNA
+ - - R/K
ASCC3
Tubulin
G.
C
C+DNB
A3+DNB
C C A A siRNA
- + - + DNB
ASCC3
Tubulin
H.
- + UVB
75
50
37
25
20
- FL

Figure S4. UVB damaged mRNA results in ASCC-resistant stalled ribosomes. Related to Figure 5. (**A)** HeLa cells were exposed to 300 J/m^2 UVB (upper) or 20 μM 4NQO (lower) to induce ribosome stalling, after which translation was inhibited with harringtonine for 5, 10, 20 and 40 minutes. Ribosome collisions were then monitored using a MNase assay to determine whether stalled ribosomes can translocate off the mRNA. **(B)** Translation was analysed using sucrose density gradient centrifugation of lysates prepared from untreated HeLa cells, cells treated with 1 μM hippuristanol (HPL) for 30 minutes, or cells that were first treated with hippuristanol and then allowed to recover for 60 minutes after the hippuristanol was removed from the media. Hippuristanol completely blocks translation initiation such that there are no polysomes. After removal of the hippuristanol there is a partial recovery of mRNA translation. **(C)** Ribosome collisions that form at sites of UVB damage after a translation initiation block is released can persist for up to 4 hours. Translation was blocked in HeLa cells with hippuristanol, after which the cells were treated with 600 J/m^2 UVB and the hippuristanol was removed from the media. Cells were harvested at 1 and 4 hours after release of the translation block and compared to cells that were released from a hippuristanol block but were not treated with UVB (UT). Collided ribosomes were detected using a MNase assay. **(D)** Comparison of the number of collided ribosomes formed at sites of UVB damage after cells were released from hippuristanol in control (ctrl) and ASCC3 (A3) depleted cells (top). Western analysis revealed that ASCC3 was efficiently depleted in these experiments (bottom). ASCC3 depletion had no effect on ribosome collisions, indicating that ribosomes stalled by UVB damage under these conditions cannot be resolved by the ASCC. **(E)** HeLa cells were grown in complete media (+R/K) or media lacking arginine and lysine (-R/K) for 6 hours. Inhibition of GCN2 (G2i) in cells depleted of arginine and lysine resulted in the accumulation of collided ribosomes. **(F)** Control (Ctrl) or ASCC3-depleted HeLa cells were grown in media lacking arginine and lysine (-R/K) for 3 hours in the presence of a GCN2 inhibitor (G2i). ASCC3 depletion resulted in increased ribosome collisions caused by arginine and lysine deficiency (top). ASCC3 is efficiently depleted in this experiment (bottom). **(G)** Treatment of HeLa cells with 0.2 μM didemnin B (DNB) resulted in ribosome stalling. Increased ribosome collisions were observed in ASCC3 (A3) depleted cells treated with didemnin B, indicating that the ASCC resolves didemnin B stalled ribosomes (top). ASCC3 was efficiently depleted in this experiment (bottom). **(H)** Translation of *in vitro* transcribed luciferase RNA treated with UVB (+) or mock-treated (-) in rabbit reticulocyte lysate. Synthesis of full-length luciferase protein (FL) decreases after UVB irradiation. Asterisks highlight truncated polypeptides that appear after UVB irradiation of the mRNA.

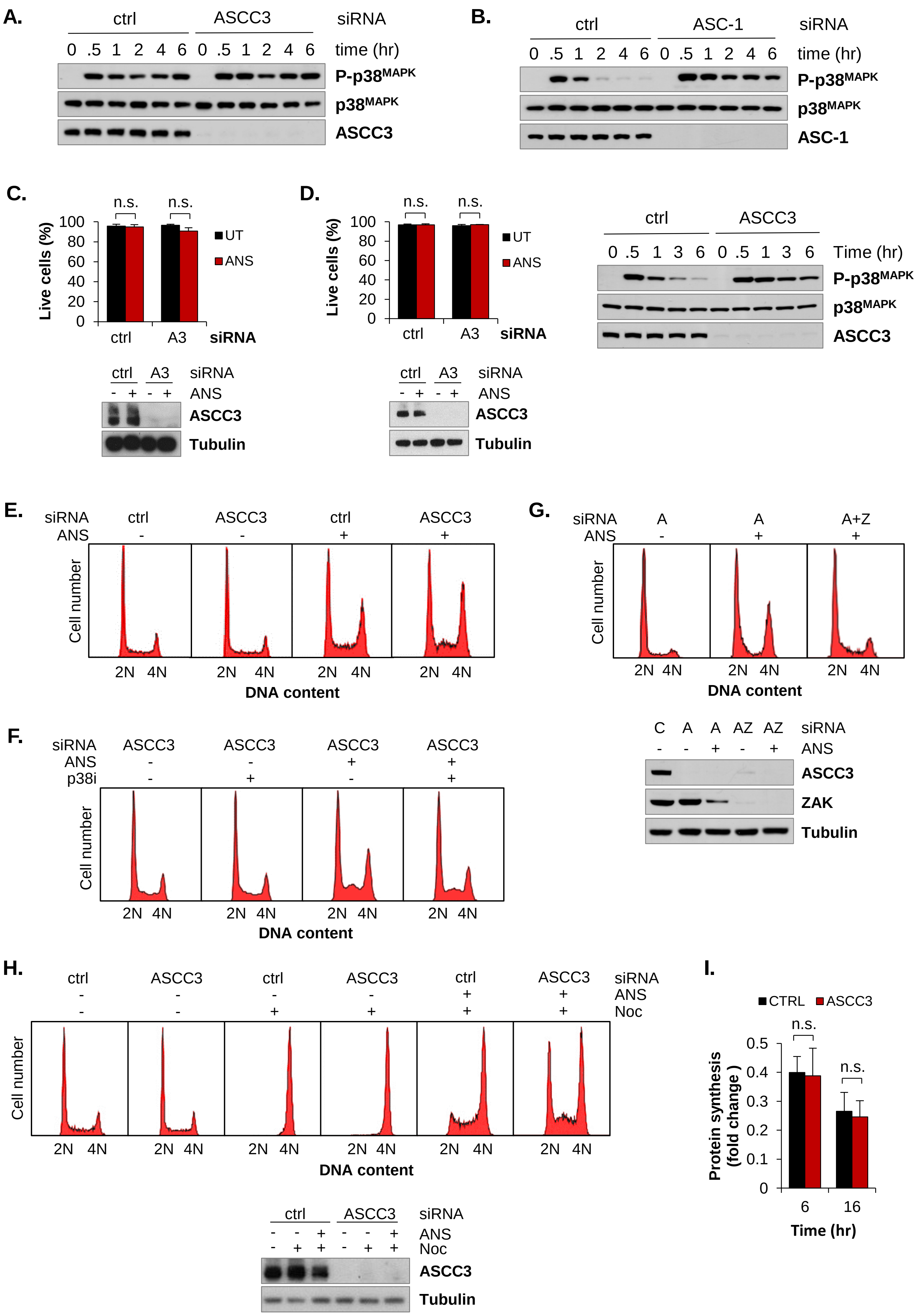
A.
ctrl
ASCC3
siRNA
0 .5 1 2 4 6 0 .5 1 2 4 6
time (hr)
P-p38MAPK
p38MAPK
ASCC3
B.
ctrl
ASC-1
siRNA
0 .5 1 2 4 6 0 .5 1 2 4 6
time (hr)
P-p38MAPK
p38MAPK
ASC-1
C.
n.s.
n.s.
Live cells (%)
100
80
60
40
20
0
UT
ANS
ctrl
A3
siRNA
ctrl
A3
siRNA
- + - +
ANS
ASCC3
Tubulin
D.
n.s.
n.s.
Live cells (%)
100
80
60
40
20
0
UT
ANS
ctrl
A3
siRNA
ctrl
A3
siRNA
- + - +
ANS
ASCC3
Tubulin
ctrl
ASCC3
0 .5 1 3 6 0 .5 1 3 6
Time (hr)
P-p38MAPK
p38MAPK
ASCC3
E.
siRNA
ANS
ctrl
ASCC3
ctrl
ASCC3
- - + +
Cell number
2N 4N
DNA content
G.
siRNA
ANS
A
A
A+Z
- + +
Cell number
2N 4N
DNA content
C A A AZ AZ
siRNA
- - + - +
ANS
ASCC3
ZAK
Tubulin
F.
siRNA
ANS
p38i
ASCC3
ASCC3
ASCC3
ASCC3
- - + +
- + - +
Cell number
2N 4N
DNA content
H.
ctrl
ASCC3
ctrl
ASCC3
ctrl
ASCC3
siRNA
- - - - + +
ANS
- - + + + +
Noc
Cell number
2N 4N
DNA content
ctrl
ASCC3
siRNA
- - + - - +
ANS
- + + - + +
Noc
ASCC3
Tubulin
I.
CTRL
ASCC3
n.s.
n.s.
Protein synthesis (fold change)
0.5
0.4
0.3
0.2
0.1
0
6
16
Time (hr)

Figure S5. Unresolved stalled ribosomes cause cell cycle arrest. Related to Figure 6.

(A) Western analysis showing that ASCC3 depletion had little effect on p38^{MAPK} phosphorylation after treatment of HeLa cells with 300 J/m^2 UVB. **(B)** Depletion of ASC-1 resulted in prolonged p38^{MAPK} phosphorylation after treatment of HeLa cells with 0.2 μM anisomycin. **(C)** Cell death was monitored in control HeLa cells (ctrl) and ASCC3-depleted (A3) cells treated with 0.2 μM anisomycin for 16 hours using Annexin V-FITC/DRAQ7 staining in three independent experiments (top). A Tukey's multiple comparison test revealed that all differences were non-significant. Thus, anisomycin had no effect on cell death in either control or ASCC3-depleted cells. Western analysis confirmed that ASCC3 was depleted efficiently in these experiments (bottom) **(D)** A similar experiment as described in C was performed on MCF10A cells. Despite sustained p38^{MAPK} signalling in response to anisomycin in ASCC3 depleted cells (right), there was no increase in cell death in this p53-proficient cell line (left). **(E)** Cell cycle profiles from one of the three-independent experiments quantified in figure 6F. **(F)** Cell cycle profiles from one of the three-independent experiments quantified in figure 6H. **(G)** Cell cycle profiles from one of the three-independent experiments quantified in figure 6I (top). Western analysis confirmed efficient depletion of ASCC3 and ZAK in these experiments (bottom) (**H)** Cell cycle profiles from one of the three independent experiments quantified in figure 6J (top). Western analysis confirmed efficient ASCC3 depletion in these experiments (bottom). **(I)** Fold change in protein synthesis in control (CTRL) and ASCC3 depleted (ASCC3) cells at 6 or 16 hours after anisomycin treatment. Anisomycin decreases protein synthesis to the same extent in both control and ASCC3-depleted cells.

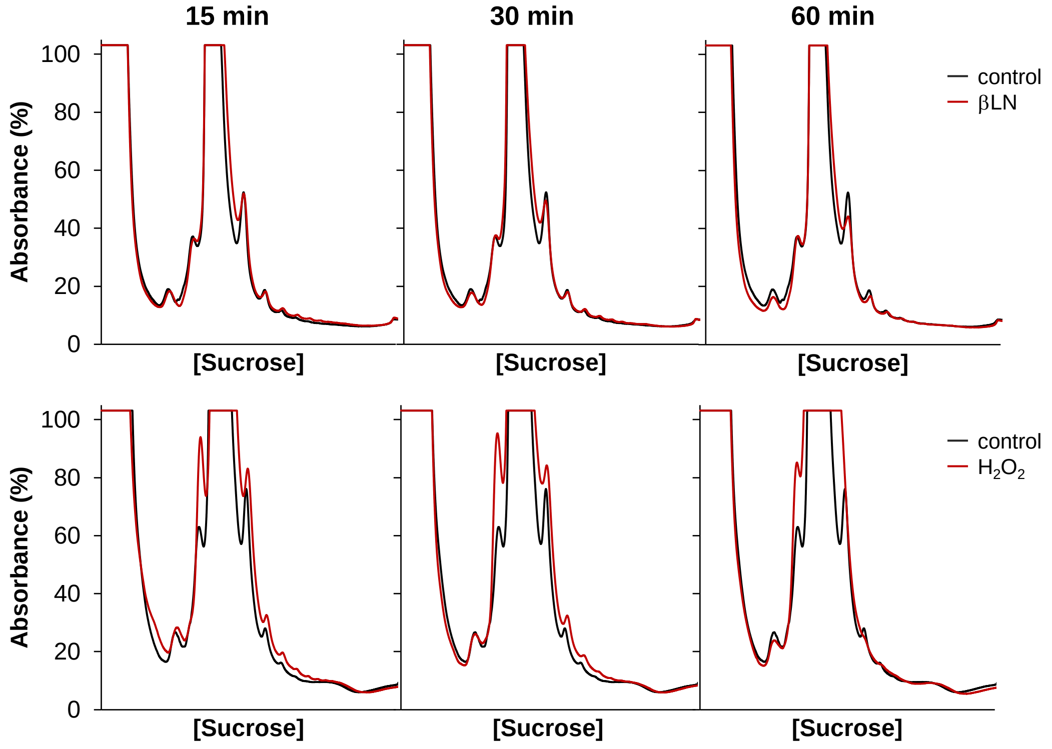

Figure S6. Unresolved stalled ribosomes do not accumulate in the cell after oxidative stress. Related to Figure 5 and the Discussion.

Collided ribosomes in HeLa cells treated with 20 μM β-lapachone (βLN) (top) or 1 mM hydrogen peroxide (bottom) for 15, 30 or 60 minutes. There was no increase in collided ribosomes in cells treated with βLN or H_2O_2, suggesting that oxidative stress does not produce unresolved stalled ribosomes in the cell.
